# The Complete Plastid Genomes of Seven Sargassaceae Species and Their Phylogenetic Analysis

**DOI:** 10.3389/fpls.2021.747036

**Published:** 2021-11-05

**Authors:** Ruoran Li, Xuli Jia, Jing Zhang, Shangang Jia, Tao Liu, Jiangyong Qu, Xumin Wang

**Affiliations:** ^1^College of Life Sciences, Yantai University, Yantai, China; ^2^College of Marine Life Sciences, Ocean University of China, Qingdao, China; ^3^School of Bioengineering, Qilu University of Technology (Shandong Academy of Sciences), Jinan, China; ^4^College of Grassland Science and Technology, China Agricultural University, Beijing, China; ^5^State Key Laboratory of Marine Environmental Science and College of Ocean and Earth Sciences, Xiamen University, Xiamen, China

**Keywords:** brown algae, co-linear analysis, comparative analysis, phylogenetic analysis, plastid genome, Sargassaceae

## Abstract

Sargassum is one of the most important genera of the family Sargassaceae in brown algae and is used to produce carrageenan, mannitol, iodine, and other economic substances. Here, seven complete plastid genomes of *Sargassum ilicifolium var. conduplicatum, S. graminifolium, S. phyllocystum, S. muticum, S. feldmannii, S. mcclurei*, and *S. henslowianum* were assembled using next-generation sequencing. The sizes of the seven circular genomes ranged from 124,258 to 124,563 bp, with two inverted regions and the same set of plastid genes, including 139 protein-coding genes (PCGs), 28 transfer (t)RNAs, and 6 ribosomal (r)RNAs. Compared with the other five available plastid genomes of Fucales, 136 PCGs were conserved, with two common ones shared with *Coccophora langsdorfii*, and one with *S. fusiforme* and *S. horneri*. The co-linear analysis identified two inversions of trnC(gca) and trnN(gtt) in ten Sargassum species, against *S. horneri* and *C. langsdorfii*. The phylogenetic analysis based on the plastid genomes of 55 brown algae (Phaeophyceae) showed four clades, whose ancient ancestor lived around 201.42 million years ago (Mya), and the internal evolutionary branches in Fucales started to be formed 92.52 Mya, while Sargassum species were divided into two subclades 14.33 Mya. Our novel plastid genomes provided evidence for the speciation of brown algae and plastid genomic evolution events.

## Introduction

The brown algal genus *Sargassum*, which belongs to the Class Phaeophyceae, Subclass Fucophycidae, Order Fucales, Family Sargassaceae, contains 360 species,^1^ accounting for 65% of the Fucales (Guiry and Guiry, 2021). The genus is mainly distributed in temperate, subtropical, and tropical habitats in the three basins of the Atlantic, Pacific, and Indian Oceans (Zhi et al., 2020). *Sargassum*, as a supplier of marine primary productivity, provides habitats, breeding grounds, and food sources for a variety of marine creatures (Godoy and Ricardo, 2002), and improves the ecological environment of the shallow sea (Komatsu et al., 2007; Williams and Feagin, 2010). Not only can it adsorb nitrogen (N), phosphorus (P), and other nutrients, but also enrich heavy metal ions in seawater (Davis et al., 2000), such as cadmium ion (Cd^2+^) (Hashim and Chu, 2004), lead ion (Pb^2+^) (Holan and Volesky, 1994; Fourest and Volesky, 1996), zinc ion (Zn^2+^) (Fourest and Volesky, 1997), nickel ion (Ni^2+^) (Holan and Volesky, 1994; Veit et al., 2009; Bermúdez et al., 2011), aluminum cation (Al^3+^) (Lee and Kamura, 1997), and arsenic ion (As^3+^) (Han et al., 2009), among other heavy metal ions. Furthermore, the various nutrient contents extracted from *Sargassum*, such as polysaccharides, are of high economic value for food, health, and medicine (Sousa et al., 2007; Ermakova et al., 2011; Liu et al., 2012). The nutrient contents also have applications in the industry. The iodine, mannitol, and algin extracted from Sargassum are important raw materials for antioxidants (Karawita et al., 2005), anti-hyperglycemic lipids (Raghavendran et al., 2010), anticarcinogens (Dias et al., 2005), anticoagulants (Zoysa et al., 2008), certain microalgae growth inhibitors (Wang et al., 2007), and other pharmaceutical products. Many studies on Sargassum have been conducted on culture techniques (Hales and Fletcher, 1990; Aaron-Amper et al., 2020), genetic diversity (Engelen et al., 2001; Cheang et al., 2010b; Sarkar et al., 2015), and phylogenetic analysis (Sung Mi Cho et al., 2012; Dixon et al., 2014; Liu et al., 2016).

The traditional classification of brown algae is mainly based on certain morphological characteristics, such as the life-history type, fertilization mode, growth mode, structure of the thallus, and plastid characteristics. Due to few morphological differences, it is difficult to identify Sargassum species based only on morphological features (Cheang et al., 2008; Mattio and Payri, 2011), especially with the naked eye (Robba et al., 2006). Therefore, the accurate classification of the *Sargassum* species has been a severe challenge. At present, as Sargassum has received increasing attention, researches based on molecular markers have been increasing (Cheang et al., 2010a; Mattio and Payri, 2011; Noormohammadi et al., 2011). Molecular phylogenetic analyses based on DNA markers have been used to determine taxa in *Sargassum* (Phillips et al., 2005; Stiger et al., 2010), including DNA fragments from the nuclei (ITS and 18SrRNA) (Mattio et al., 2015; Susilowati et al., 2015), plastids (*rbc*L and *rbc*S) (Phillips et al., 2005; Hu et al., 2017) and mitochondria (*cox*1 spacer) (Robba et al., 2006; Stiger et al., 2010). However, these previous studies were only based on a limited number of genes, and the taxonomic structure and position of the *Sargassum* are still unclear because of the lack of common genes and species. A complete plastid genome containing > 100 genes can provide plenty of DNA variations for more stable phylogenetic results.

Plastid genomes are suitable for the phylogenetic analysis of different taxonomies (Palmer, 1994), with great potential for solving phylogenetic problems due to their relatively conservative genome structure and high base substitution rate (Gaut et al., 1996; Moore et al., 2010). The plastid is a very important organelle with its own covalently closed circular double-stranded DNA and is responsible for photosynthesis. It also participates in the metabolic processes of starch, fatty acids, amino acids, pigments, and vitamins (Martin and Herrmann, 1988). The hypothetical origin of plastids was from an endogenous event in the eukaryotic host cells and cyanobacteria (Kowallik, 1994). In the theory of endosymbiosis, the ancestors of the plastids of cyanobacteria or certain photosynthetic bacteria were engulfed by primitive eukaryotic cells with powerful phagocytic ability, and symbiotically evolved into the current plastids (Palmer, 2000; McFadden, 2001; Dyall et al., 2004). The highly conserved characteristics and slow evolution rate of plastids make them an ideal tool for studying molecular evolution (Yoon et al., 2006; Verbruggen et al., 2010; Janouškovec et al., 2017). The plastids of most terrestrial plants and some algae are highly conserved in terms of genome organization, gene sequence, and essential gene content (Kato et al., 2001; De Las Rivas et al., 2002). The structure of the plastid genome can generally be divided into four regions: large single-copy region (LSC), small single-copy region (SSC), and two inverted repeat (IR) regions (IRa and IRb). The plastid genome of most advanced plants has a typical four-segment structure. The plastid genome has the remarkable feature of a pair of IRs (Wataru and Tsuneaki, 2018), but the IRs are possibly lost in the algal plastid genome (Ji et al., 2010; Wang, 2017). Comparative genomics analysis of the whole plastid genome showed higher gene content in green algae than in terrestrial plants (Simpson and Stern, 2002). The accumulation of plastid genome resources is also valuable for tracing the origin and evolution of photosynthesis in brown algae (Zhang et al., 2020), such as Sargassum. Therefore, the assembly of plastid genomes has been recognized as an efficient way to clarify phylogenetic relationships (Gao et al., 2010), and to identify plastid genome variations (Wang L. et al., 2013; Zhang et al., 2013; Liu N. et al., 2019; Liu T. et al., 2019; Liu et al., 2020).

As next-generation sequencing technology is becoming more affordable with high-throughput, more and more complete plastid genome sequencing projects have been established (Zhang et al., 2020). Only six of the currently available algal plastid genomes belong to species of the order Fucales, including one from Fucaceae (*Fucus spiralis*) and five from Sargassaceae (*Sargassum fusiforme, S. hemiphyllum var*. *chinense, S.horneri, S. thunbergii*, and *Coccophora langsdorfii*) (Yang et al., 2016; Louis et al., 2017; Cui et al., 2019; Liu et al., 2020; Zhang and Wang, 2021). Here, we assembled seven complete plastid genomes of *S. ilicifolium var*. *conduplicatum, S. graminifolium, S. phyllocystum, S. muticum, S. feldmannii, S. mcclurei*, and *S. henslowianum*. We obtained information on the gene content, genome structure, and organization of the Sargassaceae plastid genomes, and compared them with other available plastid genomes of the order Fucales and other algae. We reconstructed the phylogenetic relationship of Phaeophyceae, with an emphasis on the internal branches of the family Sargassaceae within order Fucales, and provided effective resources for molecular and phylogenetic researches on brown algae.

## Materials and Methods

### Sample Collection and DNA Extraction

Fresh thalli of *S. ilicifolium var*. *conduplicatum* (specimen number: 2015050009, Supplementary Figure 1A) from Qionghai City, Hainan Province, China (19°6′36″ N, 110°34′48″ E), *S. graminifolium* (specimen number: 2016030038, Supplementary Figure 1B) from Mirs Bay, Guangdong Province, China (22°32′58″ N, 114°34′7″ E), *S. phyllocystum* (specimen number: 2016050141, Supplementary Figure 1C) from Qionghai Base, Hainan Province, China (19°20′29″ N, 110°40′09″ E), *S. muticum* (specimen number: 2017020007, Supplementary Figure 1D) from Heishijiao, Dalian, China (38°52′7″ N, 121°33′30″ E), *S. feldmannii* (specimen number: 2017030072, Supplementary Figure 1E) from Huitou Xiaodonghai, Sanya City, Hainan Province, China (18°12′24″ N, 109°29′47″ E), *S. mcclurei* (specimen number: 2017030085, Supplementary Figure 1F) from Haiwei Port, Haiwei Town, Changjiang City, Hainan Province, China (19°26′52″ N, 108°50′9″ E), and *S. henslowianum* (specimen number: 2017050197, Supplementary Figure 1G) from Putian, Fujian Province, China (25°2′23″ N, 119°7′50″ E) were collected. The thalli were cultivated in sterilized filtered seawater under fluorescent light at 24–26°C (80–110 μmol photons m^–2^ s^–1^; 12 h light/dark cycles) and washed in autoclaved seawater before being dried with paper towels. All the samples were stored in the Culture Collection of Seaweed at the Ocean University of China.

Genomic DNA was extracted from approximately 1 g of fresh tissue using the reformative cetyl trimethyl ammonium bromide (CTAB) method (Sun et al., 2018). β-mercaptoethanol was used to suppress the oxidation of phenols, and two concentrations of CTAB (2% and 10%) were used for the removal of polysaccharides. The quality and quantity of DNA were determined using a NanoDrop ND1000 spectrophotometer (Thermo Fisher Scientific, Waltham, Massachusetts, United States).

### High-Throughput Sequencing and Assembly

Approximately 5 μg of purified DNA from each species was used to construct three short-insert libraries, according to the instructions of the manufacturer (Illumina Inc., San Diego, California, United States). Approximately 9 Gb of paired-end sequencing data was used as input in the SOAPdenovo (Luo et al., 2012), based on the default assembly parameters, to assemble the plastid genomes. The plastid genome of *S. fusiforme* (GenBank accession number: NC_048511) was used as the reference sequence to determine the proportion of the plastid-related contigs. Subsequently, all plastid-related contigs were aligned and ordered into a circular structure using the CodonCode Aligner (CodonCode Corporation, Centerville, Massachusetts, United States) (Liu N. et al., 2019).

### Annotation and Comparative Genome Analysis

The protein-encoding genes, ribosomal (r)RNA genes, and transfer (t)RNA genes of the seven species were annotated based on those of *S. fusiforme* using Geneious R10 (Biomatters Ltd., New Zealand; available on http://www.geneious.com/). The tRNA genes were predicted using tRNAscan-SE version 1.21^2^ (Schattner et al., 2005). The physical mappings of the seven plastid genomes were obtained using Organellar Genome DRAW (OGDRAW) version 1.3.1^3^ (Greiner et al., 2019). The sequence alignment and base composition were conducted using MEGA 7.0, (Sudhir et al., 2016). The plastid genomes of the seven reported species from order Fucales were aligned using the ascensive Mauve genome aligner under the default settings in the Geneious software (Drummond, 2012).

### Phylogenetic Analysis

The phylogenetic analysis of the class Phaeophyceae was conducted based on 73 shared plastid protein-encoding genes from 48 plastid genomes available in the GenBank database and the seven plastid genomes obtained in this study, with *Eunotia naegelii* (GenBank accession number: NC_024928) as the outgroup species. The protein sequences were aligned in MEGA 7.0, and the entire concatenated alignment was manually generated in BioEdit (Hall, 1999). The Gblocks server^4^ (Castresana, 2000) was used to remove the poorly conserved regions, and only 19,340 of the original 22,677 amino acids were retained in the concatenated protein alignments. To reconstruct the phylogenetic tree, the best-fitting model for the maximum-likelihood (ML) was selected using ProtTest 3.4.2 (Darriba et al., 2011), and subsequently, the phylogeny was conducted by ML bootstrap analysis using RAxML v8.2.12 (Stamatakis, 2006), with 1,000 replications under the JTT + G + F model. Bayesian inference (BI) was performed using MrBayes version 3.1.2 (Huelsenbeck and Ronquist, 2001), and the phylogenetic analysis was performed using two independent runs with four Markov chains, which ran for 1,000,000 generations until the average SD of split frequencies was below 0.01. The output trees were sampled every 100 generations. In addition, the first 25% of the samples were removed as residue, and the rest were used to build a 50% majority rule consensus tree accompanied by posterior probability values. The phylogenetic tree was plotted in FigTree version 1.3.1^5^ (Rambaut, 2009).

The MCMCTREE in the PAML4 (Yang, 2007) software package was used to estimate the species divergence time, based on the “global clock” molecular clock model and the “HKY85” nucleic acid replacement model. Additionally, a total of four calibrations were selected: *Sargassaceae* vs. *Fucaceae*, 49–66 million years ago (Mya); *Laminariaceae* vs. *Lessoniaceae*, 25.0–107.0 Mya; *Scytosiphonaceae* vs. *Ectocarpaceae*, 87.0–114.6 Mya; *Laminariales* vs. *Ectocarpales*, 129–220 Mya. The calibration points were obtained from the Timetree website^6^.

## Results

The algae in the genus Sargassum are usually yellowish or brown, with a plant height of approximately 50–100 cm, and the algae fixer is a fake root or disc. The main branches are mostly cylindrical, and the secondary branches alternate. Most of the leaves have middle ribs and large variations in shape, and most of the leaf edges are serrated. It has airbags, mostly elliptical or circular, with sub-cylindrical or leaf-like stalks, and the length stalk is variable.

### Genome Features

The seven plastid genomes were assembled as single circular molecules, with sizes ranging from 124,258 to 124,563 bp (Table 1). The average GC contents of the plastid genomes were 30.49% (*S. ilicifolium var*. *conduplicatum*), 30.43% (*S. graminifolium*), 30.42% (*S. phyllocystum*), 30.40% (*S. muticum*), 30.35% (*S. feldmannii*), 30.43% (*S. mcclurei*), and 30.44% (*S. henslowianum*), which were similar to those previously reported for Fucales species. All seven complete plastid genomes were submitted to GenBank under the following accession numbers: MW767830 (*S. ilicifolium var. conduplicatum*), MW784163 (*S. graminifolium*), MW784165 (*S. phyllocystum*), MW784166 (*S. muticum*), MW784167 (*S. feldmannii*), MW784168 (*S. mcclurei*), and MW784169 (*S. henslowianum*). The general features of the five previously reported complete plastid genomes from order Fucales, including three species from Sargassum (*S. fusiforme, S. horneri*, and *S. thunbergii*), one species from Coccophora (*C. langsdorfii*), and one species from Fucus (*F*. *spiralis*) are summarized in Table 1 for a comprehensive comparison.

**TABLE 1:** General features of the complete plastid genomes are available in the order Fucales.

	S. ilicifolium var. conduplicatum*	S. graminifolium*	S. phyllocystum*	S. muticum*	S. feldmannii*	S. mcclurei*	S. henslowianum*	*S. fusiforme#*	*S. thunbergii#*	*S. horneri#*	*C. langsdorfii#*	*F. spiralis#*
Sargassum											Coccophora	Fucus
Accession number	MW767830	MW784163	MW784165	MW784166	MW784167	MW784168	MW784169	NC_048511	NC_029134	MN265366	NC_032288	MG922855
Genome size (bp)	124414	124563	124392	124401	124258	124272	124450	124298	124592	124075	124450	125066
Overall G + C content (%)	30.49	30.43	30.42	30.40	30.35	30.43	30.44	30.4	30.4	30.6	29.8	28.9
Predicted coding sequence (%)	86.06	85.96	86.02	86.14	86.17	86.11	85.99	86.04	85.67	86.08	85.66	85.68
Pairs of gene overlaps	5	5	5	5	5	5	5	5	5	5	5	5
Total spacer size (bp)	17344	17490	17387	17247	17179	17257	17428	17349	17848	17271	17846	17913
Predicted total gene content	173	173	173	173	173	173	173	173	173	173	172	173
rRNA/tRNA/CDS content	6/28/139	6/28/139	6/28/139	6/28/139	6/28/139	6/28/139	6/28/139	6/28/139	6/28/139	6/28/139	6/27/139	6/28/139
Intron number	0	0	0	0	0	0	0	0	1	1	1	0
Start codons: ATG	135	136	135	136	135	135	135	137	136	138	137	138
Start codons: GTG	2 (*psbF,rpl3*)	2 (*psbF*,*rpl3*)	2 (*psbF*,*rpl3*)	2 (*psbF*,*rpl3*)	2 (*psbF*,*rpl3*)	2 (*psbF*,*rpl3*)	2 (*psbF*,*rpl3*)	2 (*psbF*,*rpl3*)	2 (*psbF*,*rpl3*)	1 (*psbF*)	0	1 (*psbF*)
Start codons: ATT	1 (*thiS*)	1 (*thiS*)	1 (*thiS*)	0	1 (*thiS*)	1 (*thiS*)	1 (*thiS*)	0	0	0	2 (*rps8*,*rpl3*)	0
Start codons: TTA	1 (*rps18*)	0	1 (*rps18*)	1 (*rps18*)	1 (*rps18*)	1 (*rps18*)	1 (*rps18*)	0	0	0	0	0
Start codons: TTG	0	0	0	0	0	0	0	0	1 (*rps18*)	0	0	0
Stop codons: TAA	111	110	110	107	111	111	112	110	108	111	107	113
Stop codons: TAG	24	24	23	27	24	22	23	23	25	25	26	17
Stop codons: TGA	4	5	6	5	4	6	4	6	6	3	6	9
Inverted repeat region B (IRB)	73627–79046	73694–79123	73616–79037	73597–79033	73501–78934	73502–78923	73632–79053	78758–84078	79346–84791	118661–124083	1118661–124083	79597–84838
Genes contained in the IRB	rns,trnI,trnA, rnl,rrn5	rns,trnI,trnA, rnl,rrn5	rns,trnI,trnA, rnl,rrn5	rns,trnI,trnA, rnl,rrn5	rns,trnI,trnA, rnl,rrn5	rns,trnI,trnA, rnl,rrn5	rns,trnI,trnA, rnl,rrn5	rns,trnI,trnA, rnl,rrn5	rns,trnI,trnA, rnl,rrn5	rns,trnI,trnA, rnl,rrn5	rns,trnI,trnA, rnl,rrn5	rns,trnI,trnA, rnl,rrn5
Length of IRB	5419	5429	5421	5436	5433	5421	5421	5320	5446	5309	5241	5242
Inverted repeat region A (IRB)	118995–124414	119134–124563	118971–124392	118965–124401	118825–124258	118851–124272	119029–124450	1–5320	232–5677	1–5309	39673–44913	1–5242
Genes contained in the IRA	rrn5,rnl, trnI,trnA,rns	rrn5,rnl, trnI,trnA,rns	rrn5,rnl, trnI,trnA,rns	rrn5,rnl, trnI,trnA,rns	rrn5,rnl, trnI,trnA,rns	rrn5,rnl, trnI,trnA,rns	rrn5,rnl, trnI,trnA,rns	rrn5,rnl, trnI,trnA,rns	rrn5,rnl, trnI,trnA,rns	rrn5,rnl, trnI,trnA,rns	rrn5,rnl, trnI,trnA,rns	rrn5,rnl, trnI,trnA,rns
Length of IRA	5419	5429	5421	5436	5433	5421	5421	5320	5446	5309	5423	5242
Length of small single-copy region (SSC)	39950	40012	39935	39933	39892	39929	39977	40208	40031	40142	40038	40227
Length of large single-copy region (LSC)	73626	73693	73615	73596	73500	73501	73631	73438	73669	73315	73748	74355

*Obtained in this work, # To ensure accuracy and consistency comparisons, previously published plastid genome sequences of *S. fusiforme, S. horneri, S. thunbergii, C. langsdorfii*, and *F. spiralis* were re-annotated and reanalyzed using the same method as those in the plastid genomes of *S. ilicifolium var. conduplicatum, S. graminifolium, S. phyllocystum, S. muticum, S. feldmannii, S. mcclurei*, and *S. henslowianum*.

**FIGURE 1:**
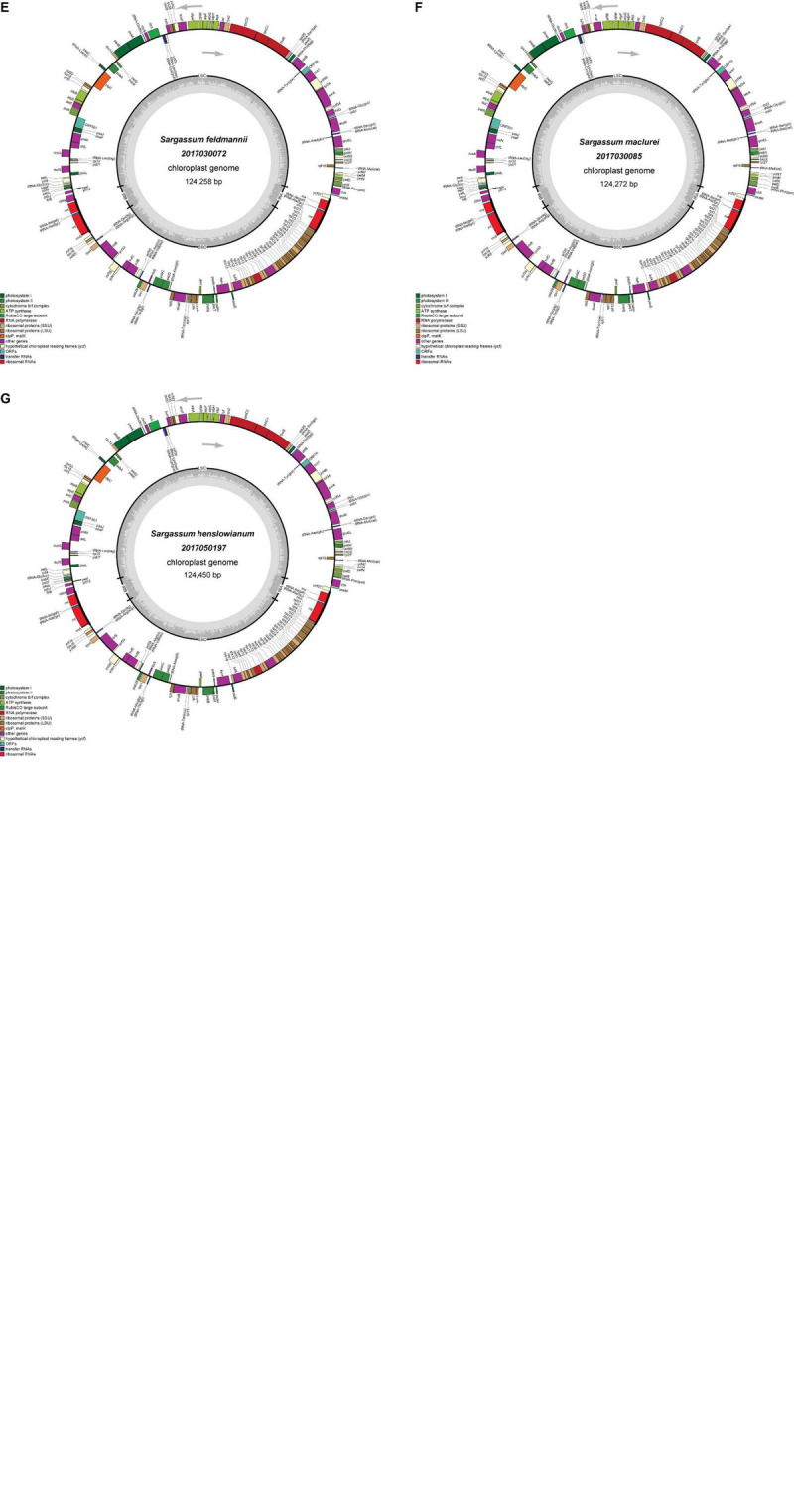
Gene maps of the plastid genomes of *S. ilicifolium var. conduplicatum* **(A)**, *S. graminifolium* **(B)**, *S. phyllocystum* **(C)**, *S. muticum* **(D)**, *S. feldmannii* **(E)**, *S. mcclurei* **(F)**, and *S. henslowianum* **(G)**. Genes on the outside of the maps are transcribed in a clockwise direction, whereas those on the inside of the maps are transcribed counterclockwise.

The seven plastid genomes were predicted to encode a total of 173 genes, including 139 protein-encoding genes, 28 tRNA genes, and 6 rRNA genes, which were the same as those of the previously reported Fucales species (Table 1). The seven genomes were conserved in the gene distribution on both the heavy and light strands (Figure 1). Most of the tRNA genes ranged from 71 to 90 bp in size, but one tRNA gene of trnL was 294 bp in size in all seven plastid genomes. For the seven plastid genomes, all tRNA and rRNA genes retained a single copy and no introns were found. However, *S. thunbergii* and *C. langsdorfii* held two IR regions (IRa and IRb) and one intron, and *S. horneri* also had one intron. In addition, similar to the majority of the Fucales species, tmRNA and miscRNA were not identified in our seven plastid genomes. In comparison with the previously published plastid genomes of the Phaeophyceae species, we confirmed that there was high conservation in the gene content and genome organization.

The coding sequence regions in the seven plastid genomes ranged from 107,070 to 107,154 bp, accounting for 86.02–86.14% of the corresponding plastid genomes. The total spacer sizes in the seven plastid genomes were 17,344, 17,490, 17,387, 17,247, 17,179, 17,257, and 17,428 bp, which were smaller than those in *S. thunbergii* (17848 bp), *C. langsdorfii* (17,846 bp), and *F. spiralis* (17,913 bp). This indicated that the seven plastid genomes from our study, together with those of *S. fusiforme* and *S. horneri* from the family Sargassum, held a relatively more compact organization. As insertions and deletions were rare, the size variations of the seven plastid genomes were mainly due to the differences in the intergenic region or non-coding region.

The single nucleotide polymorphism (SNP) sites were statistically analyzed, and the Ka/Ks values (Supplementary Table 1) were calculated to test the gene selection. Based on the analysis of 139 protein-encoding genes in the seven species, there were almost no non-synonymous changes in most of the genes, as with the value of synonymous changes of less than 1, it resulted in a final Ka/Ks value of much less than 1. Therefore, it was concluded that the genes of the seven Sargassaceae species were subject to purification selection, which suggested that in most cases, selection eliminates harmful mutations and keeps the protein unchanged. It shows that the plastid genome sequences of the seven species were relatively conservative.

The genomes of seven species in this study were compact, and there were five pairs of genes overlapping each other, where the common regions of *ftrB*-*ycf12, sufC*-*sufB, rps1*-*thiS, psbC*-*psbD*, and *rpl123*-*rpl4* were 6, 4, 4, 53, and 8 bp, respectively. These five overlaps were also found in the plastid genomes of the other Fucales species, suggesting that the five overlaps were conserved in the order Fucales. The overlapping regions of *rpl23*-*rpl4* were also observed in some plastid genomes of red algae and diatoms, indicating its high conservation in algae.

The seven plastid genomes in this study were mapped as a canonical quadripartite structure with two IRs, which divide the circular molecule into an SSC (39,892 to 40,012 bp) and an LSC (73,500 to 73,693 bp) (Figure 1). Among them, *S. phyllocystum, S. mcclurei*, and *S. henslowianum* had the same length of IRs (5,421 bp), which was different from those of *S. graminifolium* (5,429 bp), *S. feldmannii* (5433 bp), *S. muticum* (5,436 bp), and *S. ilicifolium var. conduplicatum* (5,419 bp) (Table 1).

### Protein-Encoding Genes

In terms of the predicted protein-encoding gene content, Venn diagrams of the gene contents revealed the shared and/or unique genes in the plastid genomes of the Fucales (Supplementary Figure 2). In total, the seven plastid genomes in our study encoded 139 genes, and 136 protein-encoding genes were shared by the 12 Fucales plastid genomes, accounting for 97.84% of all plastid genes. The other three genes, *orf76, orf501*, and *atpI*, were shared between the seven species in our study and *C. langsdorfii/S. horneri*, while the seven plastid genomes had lost four genes, *orf470, orf467, orf219*, and *atpl*. The atpl gene was found only in the *C. langsdorfii* plastid genome. The plastid genomes of *S. fusiforme* and *S. horneri* contained the unique genes *orf470* and *orf467*, respectively, and both covered the *orf219* gene. Several ancient genes, such as *glnB*, were absent in the plastid genomes of 12 species in Fucales.

Nearly all protein-encoding genes used ATG as the start codon in the plastid genomes of *S. ilicifolium var. conduplicatum* (135 genes, 97.12%), *S. graminifolium* (136 genes, 97.84%), *S. phyllocystum* (135 genes, 97.12%), *S. muticum* (136 genes, 97.84%), *S. feldmannii* (135 genes, 97.12%), *S. mcclurei* (135 genes, 97.12%), and *S. henslowianum* (135 genes, 97.12%). GTG, ATT, TTA, and TTG were also used as start codons in addition to ATG. GTG was used as the start codon for *psbF* and *rpl3* genes in the plastid genomes of *S. ilicifolium var. conduplicatum, S. graminifolium, S. phyllocystum, S. muticum, S. feldmannii, S. mcclurei, S. henslowianum, S. fusiform*, and *S. thunbergii*, and gene *psbF* in *S. horneri* and *F. spiralis*. ATT was used as the start codon for genes *rps8* and *rpl3* in *C. langsdorfii* and gene *thiS* in the other six genomes. The gene *rps18* used TTA as the start codon in *S. ilicifolium var. conduplicatum, S. phyllocystum, S. muticum, S. feldmannii, S. mcclurei, S. henslowianum*, and used TTG in *S. thunbergii* (Table 1).

All three typical stop codons (TAA, TAG, and TGA) were identified with an obvious preference for TAA, based on the high percentage values in the plastid genomes of *S. ilicifolium var. conduplicatum* (109, 78.42%), *S. graminifolium* (108, 77.70%), *S. phyllocystum* (108, 77.70%), *S. muticum* (106, 76.26%), *S. feldmannii* (110, 79.14%), *S. mcclurei* (110, 79.14%), and *S. henslowianum* (111, 79.86%). The *rbcR* gene used CAA as a stop codon in *S. ilicifolium var. conduplicatum* and *S. phyllocystum*.

### Comparative Analysis

**FIGURE 2:**
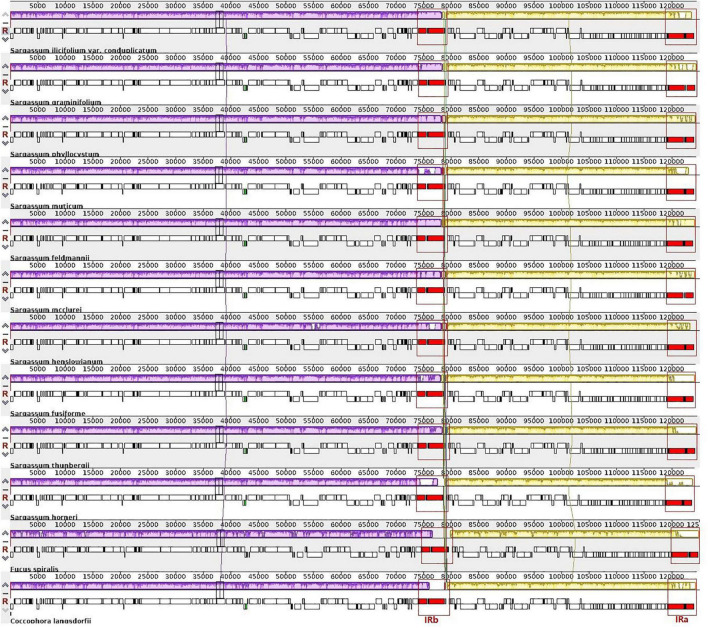
Whole-genome multiple alignments of 12 plastid genomes from the order Fucales using the Mauve software. The alignment display is organized into one horizontal “panel” per input genome sequence. Each panel of the genome contains the name of the genome sequence, a scale showing the sequence coordinates for that genome, and a single black horizontal centerline. When a block lies above the centerline, the aligned region is in the forward orientation relative to the first genome sequence. The blocks below the centerline indicate the regions that align in the reverse complement (inverse) orientation. The colored blocks in the first genome are connected by lines to the similarly colored blocks in other genomes. The area circled in red is the inverted repeat (IR) region, the purple block is the large single-copy (LSC) region, and the yellow block is the small single-copy region (SSC) region.

We further conducted a detailed co-linear analysis for the gene order in the 12 complete plastid genomes of order Fucales and confirmed strong collinearity with similar gene orders and genomic organizations (Figure 2). In Figure 2, the homologous regions are marked with similar colors and are linked by a vertical line. The white blocks indicate genomic regions with high variations, for example, the right end of the yellow blocks. Two IRs (IRa and IRb) are highlighted in red and boxed in all 12 genomes. There were no differences in gene sequences among the IR region in the Sargassum species. Two inversions covering two tRNA genes, trnC(gca) and trnN(gtt), were identified in 10 Sargassum species, against the other two Fucales species (*C. langsdorfii* and *S. horneri*). The trnC (gca) gene is on the forward strand in *S. horneri* and *C. langsdorfii*, but is on the reverse strand in the other species. The trnN (gtt) gene is forward in *S. horneri*, but is reversed in the others. The region is the only structural variation in the 12 plastid genomes, as the plastid genomes of Fucales species are highly conserved.

### Phylogenetic Analysis

**FIGURE 3:**
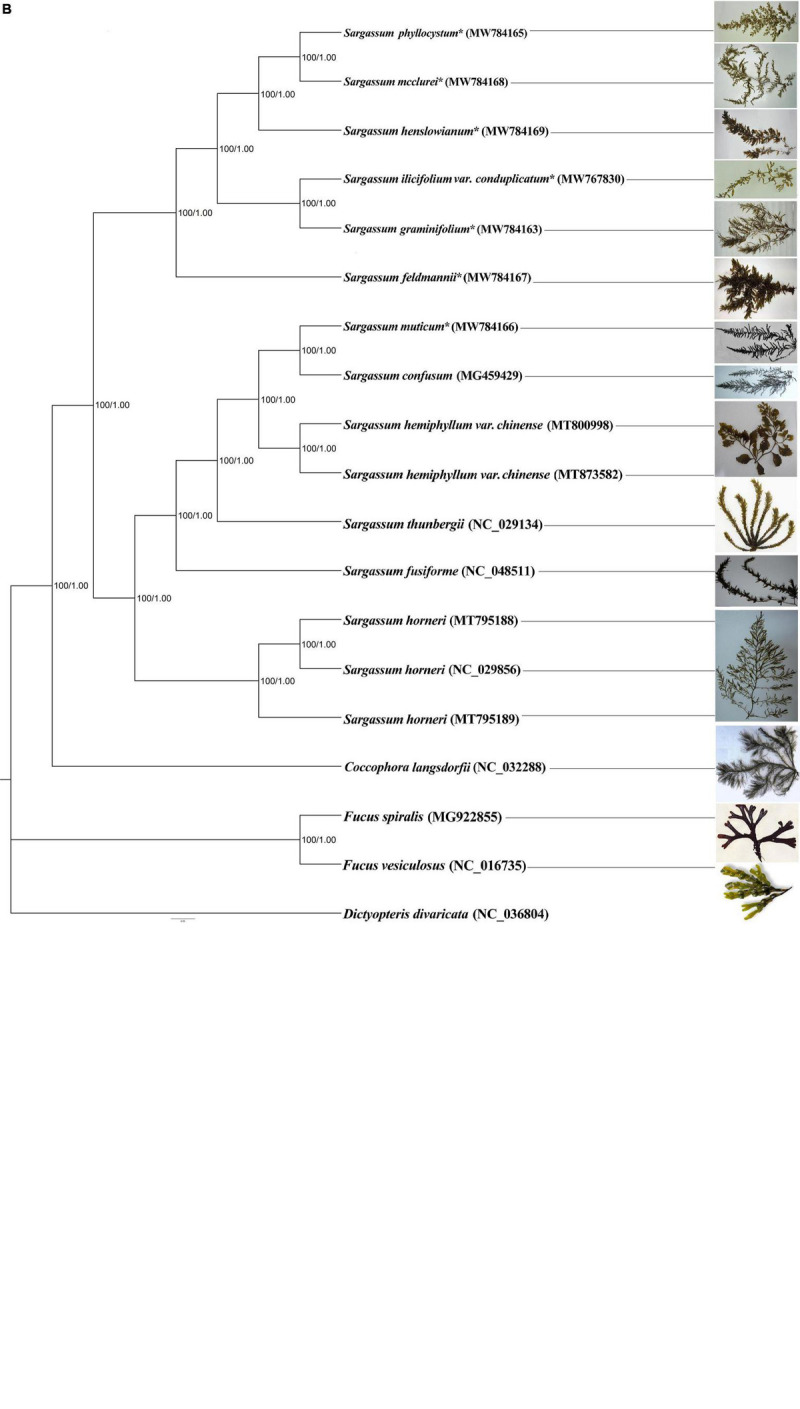
The phylogenetic tree [maximum likelihood (ML) and Bayesian inference (BI)] of 55 species from the class Phaeophyceae based on 73 shared plastid protein-encoding genes **(A)** and 18 species from the order Fucales based on 125 shared plastid protein-encoding genes **(B)**. The support values for each node are shown from maximum-likelihood bootstrap and Bayesian posterior probabilities. The asterisks indicate newly sequenced *S. ilicifolium var. conduplicatum, S. graminifolium, S. phyllocystum, S. muticum, S. feldmannii, S. mcclurei*, and *S. henslowianum* in this study.

In addition to the genus Sargassum, we downloaded the complete plastid genome sequences of the class Phaeophyceae together with the other selected algae that were available in the National Center for Biotechnology Information (NCBI), to perform phylogenetic analysis and elucidate the global phylogeny. We collected protein-encoding sequences from a total of 55 algal plastid genomes (Supplementary Table 3). Finally, we obtained 73 common genes to construct the ML and BI phylogenetic trees, with *Eunotia naegelii* as the outgroup (Figure 3A). The results showed that the topological structure of the phylogenetic trees inferred by the ML and BI methods were highly concordant and divided all species into five clades corresponding to their orders: Desmarestiales, Dictyotales, Ectocarpales, Fucales, and Laminariales. In the ML and BI trees, high bootstrap support and posterior probability values were obtained, except for the clade containing *Hapterophycus canaliculatus* and *Scytosiphon lomentaria*, and the clade containing *Egregia menziesii* and *Ecklonia arborea*.

We further performed a phylogenetic analysis based on 125 common genes of 18 species of the order Fucales, and the phylogenetic tree divided them into three clades, one for 15 *Sargassum* species, one for *Fucus*, and another for *C. langsdorfii*. Fifteen Sargassum species were divided into two subclades, and this phylogenetic tree was strictly stable with high bootstrap support and posterior probability values (Figure 3B).

### Time-Calibrated Phylogeny Analysis

**FIGURE 4:**
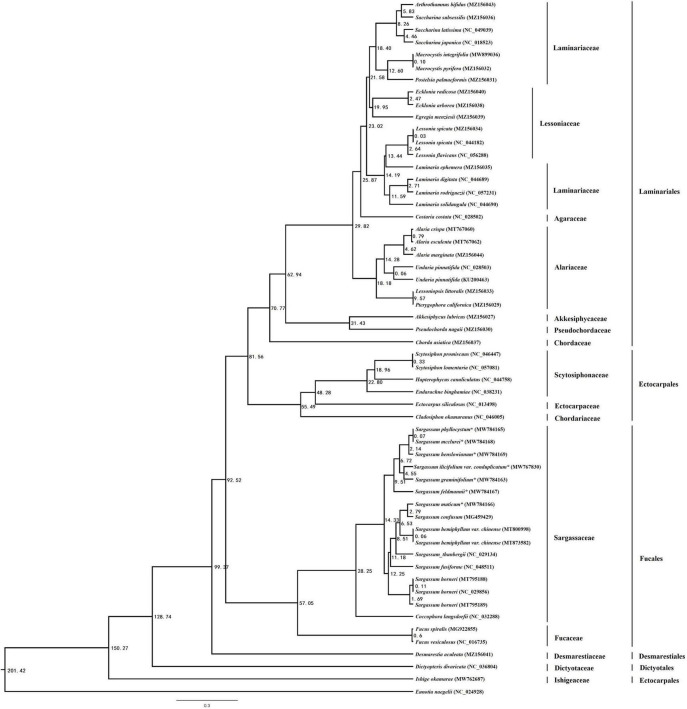
The chronogram resulting from the relaxed molecular clock analysis performed using the program MCMCtree in PAML. The time unit is 1 Mya.

The brown algae chronogram was inferred based on nucleotide sequences (Figure 4). Firstly, the Phaeophyceae node was estimated to be formed about 201.42 Mya, [with a 95% highest probability density (zHPD): 135.29–237.93 Mya]. The Fucales node was estimated to be formed about 92.52 Mya (with a 95% HPD: 62.05–109.16 Mya), and the divergence of the Sargassum node from *Coccophora langsdorfii* was 28.25 Mya (with a 95% HPD: 18.93–33.38 Mya). The divergence time of *S. ilicifolium var. conduplicatum* and *S. graminifolium* was 4.55 Mya (with a 95% HPD: 3.05–5.45 Mya), and the node of *S. feldmannii* was 9.51 Mya (with a 95% HPD: 6.37–11.27 Mya). The node of *S. phyllocystum* and *S. mcclurei* was.07 Mya (with a 95% HPD:0.03–0.12 Mya), and the neighbor node of *S. henslowianum* was 2.14 Mya (with a 95% HPD: 1.42–2.62 Mya).

## Discussion

Although Sargassum has a high species diversity and ecological importance, surprisingly, little information has been reported about its genomes. The purpose of this study was to provide the plastid genome information of the seven Sargassum species from the brown lineage and determine the phylogenetic structure of the Sargassum in the background of the available plastid pool of brown algae.

### Plastid Genomic Elements in Comparative Analysis

Generally, the plastid genome contains a typical pair of IR regions (Henry, 2005), which are often observed in the plastid genomes of advanced plants and green algae (Cao, 2015). But the IR loss has occurred in some algae, for example, *Rhodomonas salina* (Khan et al., 2007), *Chlorella vulgaris* (Wakasugi et al., 1997), *Ulva fasciata* (Melton and Lopez-Bautista, 2015), and *U. linza* (Wang et al., 2017). It has been reported that the existence of IRs increases the capacity of ribosomal genes (Bock and Knoop, 2012). The expansion and contraction of IR regions in the plant plastid genome is a common phenomenon that contributes to the plant plastid genome size (Huang et al., 2014). For example, green algae of both *Dunaliella salina* (Smith et al., 2010) and *Chlamydomonas reinhardtii* (Dron et al., 1982) contained IRs with sizes of 14.4 kb and 22.2 kb, respectively, which resulted in larger plastid genomes than the algae without IRs. To our best knowledge, the IR regions were found in the chloroplast genomes of all the brown algae. However, compared with green algae, the IR regions of brown algae are relatively small. For example, the IR sizes in *Vaucheria bursata* (Linne and Kowallik, 1992), *Phaeodactylum tricornutum, Odontella sinensis* (Secq et al., 2007), *Sargassum fusiforme* (Zhang et al., 2019), and *Sargassum thunbergii* (Yang et al., 2016) are 5.9, 6.9, 7.7, 5.3, and 5.4 kb, respectively (Supplementary Table 2). We revealed the high conservation of plastid genome structure and gene content and confirmed two IR regions (IRa and IRb) in Sargassum species (Table 1). Notably, IRa covers a part of the bases of the gene *rpl21*, while IRb covers a part of the gene *cbbx*. The protein-encoding genes of these seven species are under the pressure of purification selection, which indicates that most variations of the genes have been eliminated during the evolution process, and that the conservation is relatively high. Some studies believe that the gene conversion ability of the plastid genome is high, which can ensure the consistency and stability of the two IR region sequences and enhance the conservation of the plastid genome (Khakhlova and Bock, 2010). In comparison with the other published plastid genomes of red algae and green algae, our analysis pipeline revealed that most of the red algae have lost the IR region, and that the IR length of green algae is longer than that of brown algae (Table 1). It could be concluded that the seven species in this study experienced identical IR evolution events, which had occurred before the formation of order Fucales. This provides an updated understanding of the evolution of brown algae.

Some studies suggested that the evolution of the non-coding region duplication has obvious lineage specificity, while the coding region is conservative (Ku et al., 2013). The proportion of the repetitive sequences in the genome increases with the increase in the evolutionary level of an organism (Shu-Fen et al., 2017). These repeats contain a great deal of genetic information and play an important role in plastid genes (Cavalier-Smith, 2002). We found that the brown algal plastid genomes are relatively small in comparison with the plastid genomes of red algae (Liu et al., 2016; Yang et al., 2016; Zhang et al., 2018). After analyzing the plastid genomes of the Sargassum species, we found that there were few repeats in the coding regions, and most of the duplicates existed in the non-coding regions. Among them, three rRNAs were repeated in pairs, and 50% of the rRNAs are replicated. The plastid genomes of the seven species in this study consisted of 173 genes, of which 170 genes (6 rRNAs, 28 tRNAs, and 136 protein-encoding genes) were shared in the plastid genomes of the 12 species of order Fucales. It is worth noting that only the *C. langsdorfii* plastid genome lost the atpI gene, which is present in the plastid genome of most marine brown algae. This gene deletion might be due to gene loss or horizontal transfer to the nucleus (Feng and Shaojun, 2016). In addition, we found that some ancient genes were missing from the plastid genomes of the Sargassum.

Among all the available plastid genomes from the Fucales species, the majority of the protein-encoding plastid genes used ATG as the start codon, followed by GTG, ATT, TTA, and TTG. In addition to the standard start codon ATG, GTG was another commonly used start codon, which was mainly present in the bacteria, and the analysis of the organellar genomes revealed that several plastid and mitochondrial genes used GTG as the start codon in both red and brown algae (Corguillé et al., 2009; Wang L. et al., 2013; Wang X. et al., 2013; Zhang et al., 2013; Liu N. et al., 2019). The start codon GTG was utilized in the species of Fucales, showing a conserved evolutionary process of these genes. Usually known as an unusual start codon in eubacteria and archaea, TTG was located in a frame upstream from the originally proposed ATG start codon (Golderer et al., 1995). TTG as start codon had been found in *S. thunbergii*.

Despite the strong collinear conversation in the 12 reported plastid genomes from the order Fucales, we still identified an inversion of trnC(gca) in *S. horneri* and *C. langsdorfii*, and trnN(gtt) in *S. horneri*. This indicates the evolutionary positions of *S. horneri* and *C. langsdorfii* in the order Fucales. In plastid genomes, gene inversions have been reported among the various algae orders (Liu N. et al., 2019). Inversions and rearrangements of large fragments have been reported in red algae (Zhang et al., 2020), 30 kb inversion has been found in all living vascular plants (Raubeson and Jansen, 1992), and two transient reversals were shared in all ferns (Roper et al., 2007; Gao et al., 2009).

### Phylogenetic Analysis

The Phaeophyceae species are a group of multicellular marine eukaryotes that belong to the stramenopiles, and their plastids had originated from secondary endosymbiosis, in which a red alga was engulfed by a non-photosynthetic protist (Keeling, 2010). It was supposed that plastid genomes could provide more evolutionary information than mitochondrial genomes (Kuipeng et al., 2018), and brown algal plastid genomes appear to be highly rearranged in genome architectures (Feng and Shaojun, 2016).

According to our time-calibrated phylogeny, most brown algal orders had diversified in the timespan ranging through the Triassic period (251–119 Mya). For instance, the diversification of Fucales, Laminariales, and Ectocarpales was inferred to occur around 17.60 Mya. Brown algae have a particularly scarce fossil record (Draisma et al., 2003), and many of the fossils cannot be confidently assigned to brown algae (Clayton, 1988). This resulted in difficulty in identifying suitable algal fossils to elaborate a time-calibrated phylogeny (Verbruggen et al., 2009). Therefore, we decided to include four calibration scales that were based on fossils with clear evidence of a relationship with extant brown algal lineages. The origin of Sargassum was estimated to be more recent in the present study, i.e., 28.25 Mya (with a 95% HPD: 18.93–33.38 Mya), which was similar to the estimate of 22 Mya as the lower bound of the 95% HPD by Silberfeld et al. (2010), but was more ancient compared with the estimate of 6.7 Mya as the lower bound of the 95% HPD by Zhi et al. (2020).

Some previous studies have divided Ochrophytes algae into three branches: SI, SII, and SIII (Yang et al., 2012; Wetherbee et al., 2018; Bringloe et al., 2020), which is consistent with the results in a previous study (Bringloe et al., 2020). These phylogenetic analyses were mainly based on a few genes, such as SSU and LSU rDNA plus five proteins (Riisberg et al., 2009), and SSU rDNA plus four proteins (Yang et al., 2012). More genes are needed to construct reliable phylogenetic trees. In the Ochrophytes species, 34 plastid proteins were used to produce reliable phylogenetics (Ševèíková et al., 2015), and a collection of eukaryotic phylogenetic markers were used to assemble a phylogenomic matrix (Yang et al., 2012). The phylogenetic relationship of the Sargassum in our previous study was not well resolved based on only 13 concatenated chloroplast protein sequences (Jia et al., 2014). In our phylogenetic tree of this study, combined with the results of the co-linear analysis, we deduced that the genus Sargassum in the order Fucales had relatively significant differentiation in two subclades, which implied the possible non-monophyletic relationship of Sargassum. It also suggested that the species in the order Fucales form independent branches compared with other types of brown algae (Peters and Burkhardt, 1998). As there are still low bootstrap and posterior probability values for the individual branches with a lack of nuclear genome information, the phylogenetic relationships among the genus Sargassum could not be fully resolved based on the existing molecular data of the chloroplast genomes. In the future, the molecular phylogeny based on nuclear-encoded protein genes will provide more evidence for the evolutionary divergence of the Sargassum species, as their phenotypic differences were observed. To date, our results provide a complete and reliable maternal phylogenetic tree, and more whole genome sequences will possibly be included in further studies on phylogenetic trees and the evolution of Sargassum in brown algae.

## Data Availability Statement

The datasets presented in this study can be found in online repositories. The names of the repository/repositories and accession number(s) can be found below: https://www.ncbi.nlm.nih.gov/genbank/, MW767830; https://www.ncbi.nlm.nih. gov/genbank/, MW784163; https://www.ncbi.nlm.nih.gov/genbank/, MW784165; https://www.ncbi.nlm.nih.gov/gen bank/, MW784166; https://www.ncbi.nlm.nih.gov/genbank/, MW784167; https://www.ncbi.nlm.nih.gov/gen bank/, MW784168; https://www.ncbi.nlm.nih.gov/genbank/, MW784169.

## Author Contributions

RL: conceptualization, methodology, software, data curation, and writing – original draft. XJ: validation, software, and visualization. JZ: data curation and validation. SJ: supervision and writing – reviewing and editing. TL: writing – reviewing and editing and funding acquisition. JQ: data curation. XW: formal analysis. All authors contributed to the article and approved the submitted version.

## Conflict of Interest

The authors declare that the research was conducted in the absence of any commercial or financial relationships that could be construed as a potential conflict of interest.

## Publisher’s Note

All claims expressed in this article are solely those of the authors and do not necessarily represent those of their affiliated organizations, or those of the publisher, the editors and the reviewers. Any product that may be evaluated in this article, or claim that may be made by its manufacturer, is not guaranteed or endorsed by the publisher.

## Footnotes

1
https://www.algaebase.org/browse/taxonomy/?id=8389

2
http://lowelab.ucsc.edu/tRNAscan-SE/

3
https://chlorobox.mpimp-golm.mpg.de/OGDraw.html

4
http://phylogeny.lirmm.fr/phylo_cgi/one_task.cgi?task_type=gblocks

5
http://tree.bio.ed.ac.uk/

6
http://www.timetree.org/
