# Supplementary material for: The Complete Plastid Genomes of Seven Sargassaceae Species and Their Phylogenetic Analysis
Source: Front Plant Sci. 2021 Nov 5;12:747036. doi: 10.3389/fpls.2021.747036 (PMC8602799; doi:10.3389/fpls.2021.747036)
Supplement: Supplementary file 1 [file Data_Sheet_1.docx]

**Supplementary Materials:**

Table S1: The Ka/Ks values of 139 genes in seven Sargassaceae species

| genes | a_b | a_c | a_d | a_e | a_f | a_g | b_c | b_d | b_e | b_f | b_g | c_d | c_e | c_f | c_g | d_e | d_f | d_g | e_f | e_g | f_g |
| --- | --- | --- | --- | --- | --- | --- | --- | --- | --- | --- | --- | --- | --- | --- | --- | --- | --- | --- | --- | --- | --- |
| acsF | 0.10 | 0.06 | 0.08 | 0.11 | 0.07 | 0.06 | 0.07 | 0.08 | 0.11 | 0.07 | 0.07 | 0.06 | 0.06 | 0.00 | 0.02 | 0.07 | 0.07 | 0.05 | 0.07 | 0.07 | 0.03 |
| atpA | 0.00 | 0.01 | 0.01 | 0.01 | 0.01 | 0.01 | 0.01 | 0.01 | 0.02 | 0.01 | 0.01 | 0.01 | 0.01 | 0.00 | 0.00 | 0.01 | 0.01 | 0.01 | 0.01 | 0.01 | 0.00 |
| atpB | 0.00 | 0.00 | 0.01 | 0.00 | 0.00 | 0.00 | 0.00 | 0.01 | 0.00 | 0.00 | 0.00 | 0.01 | 0.00 | NA | 0.00 | 0.01 | 0.01 | 0.01 | 0.00 | 0.00 | 0.00 |
| atpD | 0.00 | 0.00 | 0.06 | 0.13 | 0.00 | 0.00 | 0.00 | 0.07 | 0.13 | 0.00 | 0.00 | 0.05 | 0.05 | NA | 0.00 | 0.12 | 0.05 | 0.04 | 0.05 | 0.06 | 0.00 |
| atpE | 0.00 | 0.00 | 0.00 | 0.02 | 0.00 | 0.00 | 0.00 | 0.00 | 0.03 | 0.00 | 0.00 | 0.00 | 0.02 | NA | 0.00 | 0.02 | 0.00 | 0.00 | 0.02 | 0.01 | 0.00 |
| atpF | 0.32 | 0.05 | 0.09 | 0.08 | 0.05 | 0.08 | 0.10 | 0.15 | 0.24 | 0.10 | 0.17 | 0.04 | 0.03 | NA | 0.04 | 0.07 | 0.04 | 0.05 | 0.03 | 0.03 | 0.04 |
| atpG | 0.00 | 0.07 | 0.09 | 0.03 | 0.07 | 0.06 | 0.06 | 0.08 | 0.02 | 0.06 | 0.04 | 0.07 | 0.05 | NA | 0.23 | 0.15 | 0.07 | 0.11 | 0.05 | 0.05 | 0.23 |
| atpH | 0.00 | NA | 0.00 | 0.00 | NA | NA | 0.00 | 0.00 | 0.00 | 0.00 | 0.00 | 0.00 | 0.00 | NA | NA | 0.00 | 0.00 | 0.00 | 0.00 | 0.00 | NA |
| atpI | 0.00 | 0.04 | 0.05 | 0.08 | 0.04 | 0.02 | 0.04 | 0.06 | 0.09 | 0.04 | 0.03 | 0.06 | 0.10 | 0.00 | 0.07 | 0.04 | 0.06 | 0.06 | 0.09 | 0.08 | 0.11 |
| cbbx | 0.00 | 0.04 | 0.02 | 0.02 | 0.04 | 0.02 | 0.04 | 0.02 | 0.02 | 0.04 | 0.01 | 0.04 | 0.03 | NA | 0.09 | 0.02 | 0.04 | 0.02 | 0.03 | 0.01 | 0.09 |
| ccs1 | 0.08 | 0.11 | 0.15 | 0.08 | 0.11 | 0.08 | 0.11 | 0.15 | 0.08 | 0.11 | 0.08 | 0.14 | 0.07 | NA | 0.06 | 0.11 | 0.14 | 0.12 | 0.07 | 0.06 | 0.06 |
| ccsA | 0.02 | 0.02 | 0.06 | 0.04 | 0.02 | 0.01 | 0.02 | 0.06 | 0.06 | 0.02 | 0.02 | 0.06 | 0.04 | NA | 0.00 | 0.07 | 0.06 | 0.05 | 0.04 | 0.04 | 0.00 |
| chlB | 0.02 | 0.02 | 0.02 | 0.04 | 0.02 | 0.02 | 0.02 | 0.02 | 0.05 | 0.02 | 0.01 | 0.02 | 0.03 | NA | 0.01 | 0.03 | 0.02 | 0.02 | 0.03 | 0.04 | 0.01 |
| chlI | 0.02 | 0.03 | 0.02 | 0.02 | 0.03 | 0.04 | 0.04 | 0.03 | 0.02 | 0.04 | 0.05 | 0.04 | 0.03 | NA | 0.11 | 0.04 | 0.04 | 0.04 | 0.03 | 0.04 | 0.11 |
| chlL | 0.01 | 0.02 | 0.02 | 0.02 | 0.02 | 0.02 | 0.01 | 0.02 | 0.02 | 0.01 | 0.01 | 0.02 | 0.03 | NA | 0.00 | 0.03 | 0.02 | 0.02 | 0.03 | 0.02 | 0.00 |
| chlN | 0.05 | 0.06 | 0.05 | 0.03 | 0.06 | 0.08 | 0.04 | 0.06 | 0.02 | 0.04 | 0.08 | 0.06 | 0.05 | NA | 0.04 | 0.06 | 0.06 | 0.08 | 0.05 | 0.07 | 0.04 |
| clpC | 0.01 | 0.00 | 0.02 | 0.02 | 0.00 | 0.00 | 0.01 | 0.02 | 0.02 | 0.01 | 0.01 | 0.02 | 0.02 | 0.00 | 0.00 | 0.02 | 0.02 | 0.02 | 0.02 | 0.02 | 0.00 |
| dnaB | 0.11 | 0.18 | 0.12 | 0.11 | 0.17 | 0.11 | 0.20 | 0.10 | 0.10 | 0.19 | 0.12 | 0.16 | 0.16 | NA | 0.16 | 0.11 | 0.15 | 0.13 | 0.16 | 0.13 | 0.14 |
| dnaK | 0.01 | 0.01 | 0.04 | 0.02 | 0.01 | 0.01 | 0.01 | 0.04 | 0.02 | 0.01 | 0.01 | 0.04 | 0.02 | NA | 0.03 | 0.06 | 0.04 | 0.04 | 0.02 | 0.02 | 0.03 |
| ftrB | NA | 0.13 | 0.03 | 0.14 | 0.25 | 0.13 | 0.00 | 0.00 | 0.07 | 0.13 | 0.00 | 0.00 | 0.05 | NA | NA | 0.03 | 0.02 | 0.00 | 0.10 | 0.05 | NA |
| ftsH | 0.00 | 0.01 | 0.01 | 0.01 | 0.01 | 0.01 | 0.01 | 0.01 | 0.01 | 0.01 | 0.02 | 0.02 | 0.02 | 0.00 | 0.00 | 0.00 | 0.02 | 0.02 | 0.02 | 0.02 | 0.00 |
| groEL | 0.04 | 0.02 | 0.02 | 0.01 | 0.02 | 0.02 | 0.02 | 0.02 | 0.01 | 0.02 | 0.02 | 0.02 | 0.01 | NA | 0.00 | 0.02 | 0.02 | 0.02 | 0.01 | 0.01 | 0.00 |
| ilvB | 0.06 | 0.06 | 0.07 | 0.08 | 0.06 | 0.07 | 0.08 | 0.08 | 0.10 | 0.07 | 0.08 | 0.07 | 0.10 | NA | 0.04 | 0.08 | 0.07 | 0.07 | 0.09 | 0.10 | 0.03 |
| ilvH | 0.05 | 0.06 | 0.07 | 0.07 | 0.06 | 0.05 | 0.03 | 0.05 | 0.02 | 0.03 | 0.02 | 0.06 | 0.03 | NA | 0.10 | 0.05 | 0.06 | 0.05 | 0.03 | 0.00 | 0.10 |
| ORF501 | 0.13 | 0.18 | 0.20 | 0.24 | 0.18 | 0.18 | 0.16 | 0.18 | 0.23 | 0.16 | 0.14 | 0.23 | 0.27 | NA | 0.18 | 0.23 | 0.23 | 0.21 | 0.27 | 0.24 | 0.18 |
| ORF76 | 0.28 | 0.02 | 0.06 | 0.09 | 0.02 | 0.02 | 0.07 | 0.10 | 0.17 | 0.07 | 0.12 | 0.04 | 0.05 | NA | 0.12 | 0.05 | 0.04 | 0.03 | 0.05 | 0.04 | 0.12 |
| petA | 0.10 | 0.06 | 0.06 | 0.03 | 0.06 | 0.07 | 0.03 | 0.04 | 0.01 | 0.03 | 0.04 | 0.04 | 0.01 | NA | 0.02 | 0.02 | 0.04 | 0.04 | 0.01 | 0.02 | 0.02 |
| petB | 0.00 | 0.00 | 0.03 | 0.00 | 0.00 | 0.03 | 0.00 | 0.03 | 0.00 | 0.00 | 0.02 | 0.03 | 0.00 | NA | 0.10 | 0.03 | 0.03 | 0.05 | 0.00 | 0.02 | 0.10 |
| petD | 0.00 | 0.00 | 0.00 | 0.02 | 0.00 | 0.00 | 0.00 | 0.00 | 0.02 | 0.00 | 0.00 | 0.00 | 0.02 | 0.00 | 0.00 | 0.01 | 0.00 | 0.00 | 0.02 | 0.02 | 0.00 |
| petF | 0.13 | 0.16 | 0.05 | 0.09 | 0.16 | 0.11 | 0.50 | 0.08 | 0.37 | 0.50 | 0.23 | 0.04 | 0.18 | NA | 0.09 | 0.10 | 0.04 | 0.03 | 0.18 | 0.08 | 0.09 |
| petG | NA | 0.00 | 0.00 | NA | 0.00 | 0.00 | 0.00 | 0.00 | NA | 0.00 | 0.00 | 0.00 | 0.00 | NA | NA | 0.00 | 0.00 | 0.00 | 0.00 | 0.00 | NA |
| petJ | 0.24 | 0.15 | 0.27 | 0.53 | 0.12 | 0.09 | 0.06 | 0.18 | 0.12 | 0.03 | 0.02 | 0.71 | 0.12 | NA | 0.12 | 0.36 | 0.65 | 0.44 | 0.06 | 0.04 | 0.00 |
| petL | NA | 0.00 | 0.00 | 0.00 | 0.00 | 0.00 | 0.08 | 0.10 | 0.08 | 0.08 | 0.08 | 0.00 | 0.00 | NA | NA | 0.00 | 0.00 | 0.00 | 0.00 | 0.00 | NA |
| petM | NA | NA | NA | 0.00 | NA | NA | NA | NA | 0.00 | NA | NA | NA | 0.00 | NA | NA | 0.00 | NA | NA | 0.00 | 0.00 | NA |
| petN | NA | 0.00 | NA | NA | 0.00 | 0.00 | 0.00 | NA | NA | 0.00 | 0.00 | 0.00 | 0.00 | NA | NA | NA | 0.00 | 0.00 | 0.00 | 0.00 | NA |
| psaA | 0.03 | 0.01 | 0.01 | 0.02 | 0.01 | 0.01 | 0.02 | 0.02 | 0.03 | 0.02 | 0.02 | 0.01 | 0.01 | 0.00 | 0.00 | 0.02 | 0.01 | 0.01 | 0.01 | 0.01 | 0.00 |
| psaB | 0.00 | 0.00 | 0.01 | 0.01 | 0.00 | 0.01 | 0.01 | 0.01 | 0.01 | 0.01 | 0.01 | 0.01 | 0.01 | NA | 0.01 | 0.00 | 0.01 | 0.01 | 0.01 | 0.02 | 0.01 |
| psaC | 0.00 | 0.00 | 0.02 | 0.00 | 0.00 | 0.00 | 0.00 | 0.02 | 0.00 | 0.00 | 0.00 | 0.02 | 0.00 | NA | 0.00 | 0.03 | 0.02 | 0.03 | 0.00 | 0.00 | 0.00 |
| psaD | 0.00 | 0.00 | 0.00 | 0.06 | 0.00 | 0.00 | 0.00 | 0.00 | 0.03 | 0.00 | 0.00 | 0.00 | 0.03 | NA | 0.00 | 0.03 | 0.00 | 0.00 | 0.03 | 0.03 | 0.00 |
| psaE | NA | 0.04 | 0.15 | 0.09 | 0.04 | 0.19 | 0.02 | 0.13 | 0.00 | 0.02 | 0.11 | 0.05 | 0.02 | NA | 0.04 | 0.09 | 0.05 | 0.08 | 0.02 | 0.12 | 0.04 |
| psaF | 0.00 | 0.07 | 0.08 | 0.04 | 0.07 | 0.05 | 0.08 | 0.10 | 0.05 | 0.08 | 0.07 | 0.10 | 0.09 | NA | 0.00 | 0.09 | 0.10 | 0.10 | 0.09 | 0.08 | 0.00 |
| psaI | NA | 0.00 | 0.00 | NA | 0.00 | 0.00 | 0.00 | 0.00 | NA | 0.00 | 0.00 | 0.00 | 0.00 | NA | NA | 0.00 | 0.00 | 0.00 | 0.00 | 0.00 | NA |
| psaJ | 0.00 | 0.00 | 0.00 | 0.00 | 0.00 | 0.00 | NA | 0.00 | 0.00 | NA | 0.00 | 0.00 | 0.00 | NA | 0.00 | 0.00 | 0.00 | 0.00 | 0.00 | NA | 0.00 |
| psaL | 0.06 | 0.03 | 0.02 | 0.00 | 0.03 | 0.03 | 0.09 | 0.05 | 0.03 | 0.09 | 0.12 | 0.03 | 0.02 | NA | 0.00 | 0.02 | 0.03 | 0.04 | 0.02 | 0.02 | 0.00 |
| psaM | 0.00 | 0.00 | 0.00 | 0.00 | 0.00 | 0.00 | 0.00 | 0.00 | 0.00 | 0.00 | 0.00 | 0.00 | NA | NA | NA | 0.00 | 0.00 | 0.00 | NA | NA | NA |
| psb28 | 0.06 | 0.07 | 0.03 | 0.00 | 0.07 | 0.07 | 0.14 | 0.03 | 0.07 | 0.14 | 0.14 | 0.03 | 0.07 | NA | NA | 0.03 | 0.03 | 0.03 | 0.07 | 0.07 | NA |
| psbA | 0.00 | 0.00 | 0.00 | 0.00 | 0.00 | 0.00 | 0.00 | 0.00 | 0.00 | 0.00 | 0.00 | 0.00 | 0.00 | NA | 0.00 | 0.00 | 0.00 | 0.00 | 0.00 | 0.00 | 0.00 |
| psbB | 0.00 | 0.00 | 0.00 | 0.00 | 0.00 | 0.00 | 0.00 | 0.00 | 0.00 | 0.00 | 0.00 | 0.00 | 0.00 | NA | 0.00 | 0.00 | 0.00 | 0.00 | 0.00 | 0.00 | 0.00 |
| psbC | 0.00 | 0.01 | 0.00 | 0.01 | 0.01 | 0.01 | 0.01 | 0.01 | 0.01 | 0.01 | 0.01 | 0.01 | 0.02 | NA | 0.00 | 0.01 | 0.01 | 0.01 | 0.02 | 0.02 | 0.00 |
| psbD | 0.00 | 0.02 | 0.02 | 0.00 | 0.02 | 0.02 | 0.02 | 0.02 | 0.00 | 0.02 | 0.01 | 0.01 | 0.01 | NA | 0.00 | 0.02 | 0.01 | 0.01 | 0.01 | 0.01 | 0.00 |
| psbE | 0.00 | 0.00 | 0.00 | NA | 0.00 | 0.00 | 0.00 | 0.00 | 0.00 | 0.00 | 0.00 | 0.00 | 0.00 | NA | 0.00 | 0.00 | 0.00 | 0.00 | 0.00 | 0.00 | 0.00 |
| psbF | NA | 0.00 | 0.00 | 0.00 | 0.00 | NA | 0.00 | 0.00 | 0.00 | 0.00 | NA | 0.00 | 0.00 | NA | 0.00 | 0.00 | 0.00 | 0.00 | 0.00 | 0.00 | 0.00 |
| psbH | 0.00 | 0.00 | 0.00 | 0.00 | 0.00 | 0.00 | 0.00 | 0.00 | 0.00 | 0.00 | 0.00 | 0.00 | 0.00 | NA | 0.00 | 0.00 | 0.00 | 0.00 | 0.00 | 0.00 | 0.00 |
| psbI | 0.00 | 0.00 | 0.00 | 0.00 | 0.00 | 0.00 | NA | 0.00 | NA | NA | NA | 0.00 | NA | NA | NA | 0.00 | 0.00 | 0.00 | NA | NA | NA |
| psbJ | 0.00 | 0.00 | 0.00 | NA | 0.00 | NA | 0.00 | 0.00 | 0.00 | 0.00 | 0.00 | 0.00 | 0.00 | NA | 0.00 | 0.00 | 0.00 | 0.00 | 0.00 | NA | 0.00 |
| psbK | NA | NA | NA | NA | NA | NA | NA | NA | NA | NA | NA | NA | NA | NA | NA | NA | NA | NA | NA | NA | NA |
| psbL | 0.00 | 0.00 | 0.00 | 0.00 | 0.00 | 0.00 | 0.00 | 0.00 | 0.00 | 0.00 | 0.00 | 0.00 | NA | NA | NA | 0.00 | 0.00 | 0.00 | NA | NA | NA |
| psbN | NA | 0.00 | 0.00 | 0.00 | 0.00 | 0.00 | 0.00 | 0.00 | 0.00 | 0.00 | 0.00 | 0.00 | 0.00 | NA | 0.00 | 0.00 | 0.00 | 0.00 | 0.00 | 0.00 | 0.00 |
| psbT | 0.00 | 0.00 | 0.00 | 0.00 | 0.00 | 0.00 | NA | 0.00 | 0.00 | NA | NA | 0.00 | 0.00 | NA | NA | 0.00 | 0.00 | 0.00 | 0.00 | 0.00 | NA |
| psbV | 0.04 | 0.05 | 0.06 | 0.05 | 0.05 | 0.05 | 0.02 | 0.04 | 0.02 | 0.02 | 0.02 | 0.04 | 0.00 | 0.00 | 0.00 | 0.05 | 0.04 | 0.04 | 0.00 | 0.00 | 0.00 |
| psbX | 0.00 | 0.00 | 0.00 | 0.00 | 0.00 | 0.00 | 0.00 | 0.00 | NA | 0.00 | 0.00 | 0.00 | 0.00 | NA | 0.00 | 0.00 | 0.00 | 0.00 | 0.00 | 0.00 | 0.00 |
| psbY | NA | 0.00 | 0.00 | NA | 0.00 | 0.00 | 0.00 | 0.00 | NA | 0.00 | 0.00 | 0.00 | 0.00 | NA | NA | 0.00 | 0.00 | 0.00 | 0.00 | 0.00 | NA |
| rbcL | 0.00 | 0.02 | 0.01 | 0.03 | 0.02 | 0.01 | 0.02 | 0.01 | 0.02 | 0.02 | 0.01 | 0.01 | 0.02 | NA | 0.05 | 0.03 | 0.01 | 0.00 | 0.02 | 0.03 | 0.05 |
| rbcR | 0.50 | 0.17 | 0.03 | 0.19 | 0.17 | 0.20 | 0.26 | 0.10 | 0.27 | 0.26 | 0.30 | 0.04 | 0.19 | NA | 0.41 | 0.11 | 0.06 | 0.06 | 0.22 | 0.19 | 0.42 |
| rbcS | 0.06 | 0.15 | 0.03 | 0.07 | 0.15 | 0.08 | 0.14 | 0.02 | 0.08 | 0.14 | 0.34 | 0.05 | 0.08 | NA | 0.06 | 0.08 | 0.05 | 0.04 | 0.08 | 0.05 | 0.06 |
| rpl1 | 0.08 | 0.08 | 0.12 | 0.05 | 0.08 | 0.09 | 0.10 | 0.12 | 0.04 | 0.10 | 0.08 | 0.16 | 0.04 | NA | 0.03 | 0.09 | 0.16 | 0.10 | 0.04 | 0.04 | 0.03 |
| rpl11 | 0.00 | 0.05 | 0.08 | 0.06 | 0.05 | 0.05 | 0.09 | 0.10 | 0.13 | 0.09 | 0.09 | 0.09 | 0.07 | NA | 0.00 | 0.06 | 0.09 | 0.08 | 0.07 | 0.07 | 0.00 |
| rpl12 | 0.89 | 0.08 | 0.13 | 0.15 | 0.08 | 0.11 | 0.12 | 0.18 | 0.39 | 0.12 | 0.28 | 0.06 | 0.03 | NA | 0.00 | 0.08 | 0.06 | 0.08 | 0.03 | 0.06 | 0.00 |
| rpl13 | 0.07 | 0.13 | 0.10 | 0.06 | 0.17 | 0.05 | 0.14 | 0.12 | 0.10 | 0.17 | 0.11 | 0.18 | 0.14 | NA | 0.15 | 0.17 | 0.20 | 0.12 | 0.17 | 0.10 | 0.22 |
| rpl14 | 0.00 | 0.00 | 0.03 | 0.02 | 0.00 | 0.00 | 0.00 | 0.03 | 0.02 | 0.00 | 0.00 | 0.04 | 0.02 | NA | NA | 0.01 | 0.04 | 0.04 | 0.02 | 0.02 | NA |
| rpl16 | NA | 0.09 | 0.03 | 0.00 | 0.09 | NA | 0.09 | 0.03 | 0.00 | 0.09 | NA | 0.04 | 0.02 | NA | 0.00 | 0.02 | 0.04 | 0.05 | 0.02 | 0.02 | 0.00 |
| rpl18 | 0.00 | 0.02 | 0.08 | 0.09 | 0.02 | 0.03 | 0.04 | 0.15 | 0.30 | 0.04 | 0.07 | 0.04 | 0.09 | NA | 0.14 | 0.09 | 0.04 | 0.11 | 0.09 | 0.14 | 0.14 |
| rpl19 | 0.06 | 0.07 | 0.08 | 0.07 | 0.07 | 0.04 | 0.03 | 0.05 | 0.05 | 0.03 | 0.00 | 0.06 | 0.06 | NA | 0.18 | 0.12 | 0.06 | 0.04 | 0.06 | 0.04 | 0.18 |
| rpl2 | 0.14 | 0.04 | 0.03 | 0.04 | 0.04 | 0.03 | 0.05 | 0.03 | 0.05 | 0.05 | 0.04 | 0.05 | 0.06 | NA | 0.08 | 0.02 | 0.05 | 0.04 | 0.06 | 0.05 | 0.08 |
| rpl20 | 0.43 | 0.33 | 0.12 | 0.32 | 0.33 | 0.37 | 0.21 | 0.07 | 0.25 | 0.21 | 0.24 | 0.08 | 0.19 | NA | 0.00 | 0.09 | 0.08 | 0.08 | 0.19 | 0.19 | 0.00 |
| rpl21 | 0.09 | 0.04 | 0.00 | 0.00 | 0.04 | 0.06 | 0.09 | 0.02 | NA | 0.09 | 0.17 | 0.03 | 0.06 | NA | 0.00 | 0.00 | 0.03 | 0.03 | 0.06 | 0.11 | 0.00 |
| rpl22 | 0.00 | 0.10 | 0.09 | 0.08 | 0.10 | 0.16 | 0.12 | 0.08 | 0.06 | 0.12 | 0.14 | 0.06 | 0.09 | NA | 0.08 | 0.05 | 0.06 | 0.08 | 0.09 | 0.13 | 0.08 |
| rpl23 | 0.00 | 0.07 | 0.06 | 0.00 | 0.07 | 0.05 | 0.03 | 0.07 | 0.00 | 0.03 | 0.04 | 0.08 | 0.04 | NA | 0.00 | 0.10 | 0.08 | 0.10 | 0.04 | 0.05 | 0.00 |
| rpl24 | 0.19 | 0.05 | 0.23 | 0.07 | 0.05 | 0.06 | 0.00 | 0.10 | 0.03 | 0.00 | 0.00 | 0.07 | 0.03 | NA | 0.00 | 0.10 | 0.07 | 0.08 | 0.03 | 0.03 | 0.00 |
| rpl27 | 0.08 | 0.00 | 0.02 | 0.00 | 0.00 | 0.00 | 0.07 | 0.08 | 0.10 | 0.07 | 0.08 | 0.01 | 0.00 | NA | 0.00 | 0.02 | 0.01 | 0.02 | 0.00 | 0.00 | 0.00 |
| rpl29 | NA | 0.06 | 0.04 | NA | 0.06 | 0.12 | 0.00 | 0.00 | NA | 0.00 | 0.00 | 0.00 | 0.06 | NA | 0.00 | 0.04 | 0.00 | 0.00 | 0.06 | 0.12 | 0.00 |
| rpl3 | 0.08 | 0.09 | 0.10 | 0.13 | 0.09 | 0.12 | 0.12 | 0.14 | 0.24 | 0.12 | 0.22 | 0.13 | 0.17 | NA | 0.00 | 0.15 | 0.13 | 0.18 | 0.17 | 0.26 | 0.00 |
| rpl31 | 0.00 | 0.03 | 0.02 | 0.08 | 0.03 | 0.00 | 0.04 | 0.02 | 0.15 | 0.04 | 0.00 | 0.05 | 0.24 | NA | 0.16 | 0.03 | 0.05 | 0.03 | 0.24 | 0.32 | 0.16 |
| rpl32 | NA | NA | 0.05 | 0.34 | NA | NA | NA | 0.05 | 0.10 | NA | NA | 0.04 | 0.00 | NA | NA | 0.06 | 0.04 | 0.04 | 0.00 | 0.00 | NA |
| rpl33 | 0.10 | 0.11 | 0.15 | 0.10 | 0.11 | 0.23 | 0.05 | 0.16 | 0.04 | 0.05 | 0.09 | 0.14 | 0.08 | NA | 0.00 | 0.26 | 0.14 | 0.18 | 0.08 | 0.11 | 0.00 |
| rpl34 | 0.06 | 0.06 | 0.07 | 0.23 | 0.06 | 0.06 | 0.12 | 0.12 | 0.21 | 0.12 | 0.12 | 0.13 | 0.05 | NA | NA | 0.12 | 0.13 | 0.13 | 0.05 | 0.05 | NA |
| rpl35 | NA | 0.00 | 0.16 | 0.06 | 0.00 | 0.20 | 0.00 | 0.16 | 0.06 | 0.00 | 0.20 | 0.19 | 0.09 | NA | NA | 0.14 | 0.19 | 0.27 | 0.09 | 0.19 | NA |
| rpl36 | NA | NA | 0.00 | 0.00 | NA | NA | NA | 0.00 | 0.00 | NA | NA | 0.23 | 0.12 | NA | NA | 0.00 | 0.23 | 0.00 | 0.12 | 0.00 | NA |
| rpl4 | 0.10 | 0.02 | 0.09 | 0.19 | 0.01 | 0.06 | 0.10 | 0.13 | 0.80 | 0.09 | 0.15 | 0.09 | 0.15 | 0.00 | 0.10 | 0.13 | 0.09 | 0.11 | 0.14 | 0.20 | 0.08 |
| rpl5 | 0.03 | 0.02 | 0.03 | 0.05 | 0.02 | 0.03 | 0.05 | 0.07 | 0.05 | 0.05 | 0.05 | 0.05 | 0.06 | NA | 0.00 | 0.09 | 0.05 | 0.05 | 0.06 | 0.06 | 0.00 |
| rpl6 | 0.22 | 0.27 | 0.06 | 0.09 | 0.27 | 0.26 | 0.18 | 0.06 | 0.06 | 0.18 | 0.18 | 0.08 | 0.13 | NA | 0.07 | 0.07 | 0.08 | 0.09 | 0.13 | 0.14 | 0.07 |
| rpl9 | 0.31 | 0.14 | 0.35 | 0.15 | 0.17 | 0.14 | 0.12 | 0.35 | 0.10 | 0.15 | 0.12 | 0.27 | 0.04 | NA | 0.00 | 0.10 | 0.30 | 0.38 | 0.05 | 0.05 | 0.05 |
| rpoA | 0.21 | 0.20 | 0.16 | 0.26 | 0.20 | 0.22 | 0.28 | 0.20 | 0.50 | 0.28 | 0.34 | 0.17 | 0.32 | NA | 0.30 | 0.18 | 0.17 | 0.20 | 0.32 | 0.38 | 0.30 |
| rpoB | 0.05 | 0.05 | 0.04 | 0.03 | 0.05 | 0.06 | 0.05 | 0.04 | 0.03 | 0.05 | 0.07 | 0.04 | 0.04 | NA | 0.07 | 0.04 | 0.04 | 0.05 | 0.04 | 0.05 | 0.07 |
| rpoC1 | 0.06 | 0.04 | 0.07 | 0.06 | 0.04 | 0.05 | 0.05 | 0.08 | 0.09 | 0.05 | 0.06 | 0.06 | 0.06 | NA | 0.00 | 0.08 | 0.06 | 0.07 | 0.06 | 0.06 | 0.00 |
| rpoC2 | 0.11 | 0.13 | 0.09 | 0.11 | 0.13 | 0.13 | 0.10 | 0.07 | 0.07 | 0.10 | 0.09 | 0.09 | 0.09 | NA | 0.17 | 0.07 | 0.09 | 0.08 | 0.09 | 0.09 | 0.17 |
| rps1 | 0.09 | 0.19 | 0.06 | 0.04 | 0.19 | 0.07 | 0.20 | 0.03 | 0.05 | 0.20 | 0.04 | 0.10 | 0.13 | NA | 0.09 | 0.05 | 0.10 | 0.06 | 0.13 | 0.06 | 0.09 |
| rps10 | 0.11 | 0.21 | 0.05 | 0.04 | 0.21 | 0.11 | 0.11 | 0.03 | 0.00 | 0.11 | 0.00 | 0.05 | 0.04 | NA | NA | 0.03 | 0.05 | 0.03 | 0.04 | 0.00 | NA |
| rps11 | 0.00 | 0.00 | 0.04 | 0.00 | 0.00 | 0.03 | 0.00 | 0.04 | 0.00 | 0.00 | 0.03 | 0.03 | 0.00 | NA | 0.21 | 0.04 | 0.03 | 0.04 | 0.00 | 0.02 | 0.21 |
| rps12 | 0.00 | 0.00 | 0.00 | 0.00 | 0.00 | 0.00 | 0.00 | 0.00 | 0.00 | 0.00 | 0.00 | 0.00 | 0.00 | NA | 0.00 | 0.00 | 0.00 | 0.00 | 0.00 | 0.00 | 0.00 |
| rps13 | 0.03 | 0.07 | 0.02 | 0.05 | 0.07 | 0.07 | 0.18 | 0.06 | 0.13 | 0.18 | 0.06 | 0.11 | 0.23 | NA | 0.00 | 0.08 | 0.11 | 0.10 | 0.23 | 0.17 | 0.00 |
| rps14 | 0.12 | 0.09 | 0.08 | 0.03 | 0.09 | 0.07 | 0.11 | 0.10 | 0.04 | 0.11 | 0.09 | 0.10 | 0.04 | NA | 0.00 | 0.10 | 0.10 | 0.09 | 0.04 | 0.04 | 0.00 |
| rps16 | 0.49 | 0.76 | 0.16 | 0.16 | 0.76 | 0.76 | 0.12 | 0.04 | 0.00 | 0.12 | 0.12 | 0.08 | 0.06 | NA | NA | 0.04 | 0.08 | 0.08 | 0.06 | 0.06 | NA |
| rps17 | 0.16 | 0.00 | 0.08 | 0.12 | 0.00 | 0.00 | 0.05 | 0.04 | 0.06 | 0.05 | 0.08 | 0.06 | 0.07 | NA | 0.00 | 0.07 | 0.06 | 0.07 | 0.07 | 0.09 | 0.00 |
| rps18 | 0.00 | 0.05 | 0.02 | 0.06 | 0.05 | 0.04 | 0.06 | 0.02 | 0.07 | 0.06 | 0.05 | 0.02 | 0.08 | NA | 0.19 | 0.03 | 0.02 | 0.05 | 0.08 | 0.14 | 0.19 |
| rps19 | NA | NA | 0.02 | 0.00 | NA | 0.00 | NA | 0.02 | 0.00 | NA | 0.00 | 0.02 | 0.00 | NA | 0.00 | 0.02 | 0.02 | 0.02 | 0.00 | 0.00 | 0.00 |
| rps2 | 0.03 | 0.07 | 0.02 | 0.07 | 0.07 | 0.04 | 0.05 | 0.01 | 0.07 | 0.05 | 0.02 | 0.03 | 0.06 | NA | 0.06 | 0.03 | 0.03 | 0.02 | 0.06 | 0.04 | 0.06 |
| rps20 | 0.42 | 0.23 | 0.36 | 0.16 | 0.23 | 0.27 | 0.32 | 0.49 | 0.28 | 0.32 | 0.39 | 0.52 | 0.10 | NA | 0.00 | 0.34 | 0.52 | 0.64 | 0.10 | 0.14 | 0.00 |
| rps3 | 0.04 | 0.04 | 0.02 | 0.06 | 0.04 | 0.05 | 0.02 | 0.02 | 0.05 | 0.02 | 0.04 | 0.02 | 0.05 | NA | 0.00 | 0.04 | 0.02 | 0.02 | 0.05 | 0.07 | 0.00 |
| rps4 | 0.20 | 0.07 | 0.10 | 0.13 | 0.07 | 0.05 | 0.04 | 0.08 | 0.15 | 0.04 | 0.03 | 0.08 | 0.18 | NA | 0.05 | 0.06 | 0.08 | 0.07 | 0.18 | 0.13 | 0.05 |
| rps5 | 0.05 | 0.03 | 0.07 | 0.02 | 0.03 | 0.04 | 0.01 | 0.07 | 0.00 | 0.01 | 0.01 | 0.04 | 0.01 | NA | 0.00 | 0.06 | 0.04 | 0.04 | 0.01 | 0.01 | 0.00 |
| rps7 | 0.05 | 0.02 | 0.03 | 0.05 | 0.02 | 0.03 | 0.04 | 0.05 | 0.07 | 0.04 | 0.05 | 0.01 | 0.01 | NA | 0.00 | 0.00 | 0.01 | 0.01 | 0.01 | 0.02 | 0.00 |
| rps8 | 0.06 | 0.04 | 0.03 | 0.12 | 0.04 | 0.04 | 0.00 | 0.00 | 0.10 | 0.00 | 0.00 | 0.00 | 0.06 | NA | 0.00 | 0.05 | 0.00 | 0.00 | 0.06 | 0.06 | 0.00 |
| rps9 | 0.10 | 0.11 | 0.01 | 0.10 | 0.11 | 0.22 | 0.03 | 0.00 | 0.04 | 0.03 | 0.07 | 0.01 | 0.07 | NA | 0.61 | 0.02 | 0.01 | 0.02 | 0.07 | 0.12 | 0.61 |
| secA | 0.06 | 0.06 | 0.07 | 0.07 | 0.05 | 0.06 | 0.05 | 0.06 | 0.06 | 0.04 | 0.05 | 0.06 | 0.06 | NA | 0.06 | 0.06 | 0.06 | 0.07 | 0.06 | 0.07 | 0.05 |
| secY | 0.11 | 0.09 | 0.10 | 0.08 | 0.09 | 0.08 | 0.10 | 0.11 | 0.10 | 0.11 | 0.10 | 0.09 | 0.08 | 0.00 | 0.00 | 0.09 | 0.09 | 0.09 | 0.08 | 0.07 | 0.00 |
| sufB | 0.03 | 0.09 | 0.01 | 0.03 | 0.09 | 0.05 | 0.09 | 0.01 | 0.03 | 0.09 | 0.05 | 0.04 | 0.07 | NA | 0.14 | 0.02 | 0.04 | 0.02 | 0.07 | 0.04 | 0.14 |
| sufC | 0.12 | 0.03 | 0.05 | 0.08 | 0.03 | 0.03 | 0.06 | 0.03 | 0.05 | 0.06 | 0.06 | 0.04 | 0.05 | NA | 0.00 | 0.04 | 0.04 | 0.04 | 0.05 | 0.05 | 0.00 |
| tatC | 0.14 | 0.07 | 0.05 | 0.09 | 0.07 | 0.04 | 0.09 | 0.05 | 0.11 | 0.09 | 0.05 | 0.06 | 0.07 | NA | 0.05 | 0.04 | 0.06 | 0.05 | 0.07 | 0.05 | 0.05 |
| thiG | 0.04 | 0.08 | 0.08 | 0.06 | 0.08 | 0.09 | 0.07 | 0.07 | 0.07 | 0.07 | 0.07 | 0.06 | 0.08 | NA | 0.00 | 0.05 | 0.06 | 0.06 | 0.08 | 0.08 | 0.00 |
| thiS | 0.07 | 0.26 | 0.10 | 0.23 | 0.26 | 0.40 | 1.05 | 0.12 | 0.64 | 1.05 | NA | 0.18 | 0.61 | NA | 0.20 | 0.10 | 0.18 | 0.16 | 0.61 | 1.35 | 0.20 |
| tsf | 0.12 | 0.06 | 0.08 | 0.19 | 0.06 | 0.11 | 0.02 | 0.05 | 0.07 | 0.02 | 0.03 | 0.05 | 0.07 | NA | 0.18 | 0.10 | 0.05 | 0.06 | 0.07 | 0.05 | 0.18 |
| tufA | 0.00 | 0.00 | 0.01 | 0.00 | 0.00 | 0.00 | 0.00 | 0.01 | 0.00 | 0.00 | 0.00 | 0.01 | 0.00 | NA | 0.00 | 0.01 | 0.01 | 0.01 | 0.00 | 0.00 | 0.00 |
| ycf12 | 0.00 | NA | NA | NA | NA | NA | 0.00 | 0.00 | 0.00 | 0.00 | 0.00 | NA | NA | NA | NA | NA | NA | NA | NA | NA | NA |
| ycf19 | 0.00 | 0.00 | 0.02 | 0.04 | 0.00 | 0.00 | 0.00 | 0.04 | 0.11 | 0.00 | 0.00 | 0.03 | 0.11 | NA | 0.00 | 0.02 | 0.03 | 0.03 | 0.11 | 0.11 | 0.00 |
| ycf3 | 0.00 | 0.00 | 0.02 | 0.00 | 0.00 | 0.00 | 0.00 | 0.02 | 0.00 | 0.00 | 0.00 | 0.02 | 0.00 | NA | NA | 0.02 | 0.02 | 0.02 | 0.00 | 0.00 | NA |
| ycf33 | NA | NA | 1.57 | 0.80 | NA | 0.22 | NA | 0.71 | 0.44 | NA | 0.00 | 0.71 | 0.44 | NA | 0.00 | 0.40 | 0.71 | 0.22 | 0.44 | 0.14 | 0.00 |
| ycf34 | 0.21 | 0.14 | 0.49 | 0.17 | 0.14 | 0.24 | 0.07 | 0.58 | 0.16 | 0.07 | 0.23 | 0.17 | 0.08 | NA | 0.51 | 0.19 | 0.17 | 0.23 | 0.08 | 0.14 | 0.51 |
| ycf35 | 0.18 | 0.07 | 0.07 | 0.13 | 0.07 | 0.08 | 0.13 | 0.16 | 0.29 | 0.13 | 0.12 | 0.07 | 0.16 | NA | 0.00 | 0.07 | 0.07 | 0.07 | 0.16 | 0.16 | 0.00 |
| ycf37 | 0.43 | 0.07 | 0.33 | 0.13 | 0.07 | 0.10 | 0.20 | 0.41 | 0.22 | 0.20 | 0.19 | 0.32 | 0.16 | NA | 0.10 | 0.41 | 0.32 | 0.37 | 0.16 | 0.14 | 0.10 |
| ycf39 | 0.11 | 0.06 | 0.05 | 0.07 | 0.06 | 0.05 | 0.04 | 0.04 | 0.06 | 0.04 | 0.03 | 0.04 | 0.07 | NA | 0.09 | 0.06 | 0.04 | 0.04 | 0.07 | 0.05 | 0.09 |
| ycf4 | 0.04 | 0.07 | 0.09 | 0.04 | 0.07 | 0.05 | 0.05 | 0.08 | 0.02 | 0.05 | 0.04 | 0.05 | 0.02 | NA | 0.00 | 0.08 | 0.05 | 0.04 | 0.02 | 0.01 | 0.00 |
| ycf41 | 0.24 | 0.12 | 0.37 | 0.63 | 0.12 | 0.25 | 0.06 | 0.32 | 0.25 | 0.06 | 0.14 | 0.23 | 0.20 | NA | 0.12 | 0.21 | 0.23 | 0.23 | 0.20 | 0.23 | 0.12 |
| ycf42 | 0.04 | 0.02 | 0.03 | 0.05 | 0.02 | 0.06 | 0.00 | 0.02 | 0.03 | 0.00 | 0.04 | 0.02 | 0.02 | NA | 0.07 | 0.01 | 0.02 | 0.04 | 0.02 | 0.05 | 0.07 |
| ycf46 | 0.03 | 0.03 | 0.02 | 0.02 | 0.03 | 0.04 | 0.01 | 0.01 | 0.01 | 0.01 | 0.02 | 0.01 | 0.02 | NA | 0.05 | 0.01 | 0.01 | 0.02 | 0.02 | 0.03 | 0.05 |
| ycf47 | NA | 0.23 | 0.00 | 0.16 | 0.23 | 0.23 | 0.21 | 0.06 | 0.14 | 0.21 | 0.22 | 0.04 | 0.08 | NA | NA | 0.06 | 0.04 | 0.04 | 0.08 | 0.09 | NA |
| ycf54 | NA | 0.11 | 0.04 | 0.11 | 0.11 | 0.23 | 0.05 | 0.07 | 0.06 | 0.05 | 0.14 | 0.07 | 0.05 | NA | 0.06 | 0.06 | 0.07 | 0.09 | 0.05 | 0.11 | 0.06 |
| ycf65 | 0.07 | 0.11 | 0.11 | 0.07 | 0.11 | 0.11 | 0.04 | 0.07 | 0.00 | 0.04 | 0.04 | 0.08 | 0.06 | NA | NA | 0.11 | 0.08 | 0.08 | 0.06 | 0.06 | NA |
| ycf66 | NA | 0.00 | 0.00 | 0.24 | 0.00 | 0.00 | 0.00 | 0.00 | 0.24 | 0.00 | 0.00 | 0.00 | 0.03 | NA | 0.00 | 0.04 | 0.00 | 0.00 | 0.03 | 0.05 | 0.00 |

The Ka/Ks values of 139 genes in seven Sargassaceae species: *Sargassum ilicifolium var. conduplicatum* (a), *Sargassum graminifolium* (b), *Sargassum phyllocystum* (c), *Sargassum muticum* (d), *Sargassum feldmannii* (e), *Sargassum mcclurei* (f), and *Sargassum henslowianum* (g).

Table S2: Structure of the inverted repeat regions of Fucales and green algae

|  | *Sargassum*  *fusiforme* | *Sargassum*  *thunbergii* | *Sargassum*  *horneri* | *Coccophora langsdorfii* | *Fucus vesiculosus*  *var. spiralis* | *Chlamydomonas*  *leiostraca* | *Chlamydomonas*  *reinhardtii* |
| --- | --- | --- | --- | --- | --- | --- | --- |
| Inverted repeat region A (IRA) start | 1 | 232 | 1 | 39673 | 1 | 5967 | 34227 |
| Inverted repeat region A (IRA) end | 5320 | 5677 | 5309 | 44913 | 5242 | 19028 | 56448 |
| Genes contained in the IRA | rns,trnI,trnA,  rnl,rrn5 | rns,trnI,trnA,  rnl,rrn5 | rns,trnI,trnA,  rnl,rrn5 | rns,trnI,trnA,  rnl,rrn5 | rns,trnI,trnA,  rnl,rrn5 | rrn5,trnI,trnA,  rrnL,rrn5,psbA | rrnS,trnI,trnA,rrn7,  rrn3,rrnL,rrn5,psbA |
| Total length of IRA | 5320 | 5446 | 5309 | 5241 | 5242 | 13062 | 22222 |
| Inverted repeat region B (IRB) start | 78758 | 79346 | 78624 | 118661 | 79597 | 83209 | 137755 |
| Inverted repeat region B (IRB) end | 84078 | 84791 | 83932 | 124083 | 84838 | 96259 | 159966 |
| Genes contained in the IRB | rns,trnI,trnA,  rnl,rrn5 | rns,trnI,trnA,  rnl,rrn5 | rns,trnI,trnA,  rnl,rrn5 | rns,trnI,trnA,  rnl,rrn5 | rns,trnI,trnA,  rnl,rrn5 | rrn5,trnI,trnA,  rrnL,rrn5,psbA | rrnS,trnI,trnA,rrn7,  rrn3,rrnL,rrn5,psbA |
| Total length of IRB | 5320 | 5446 | 5309 | 5423 | 5242 | 13051 | 22212 |

Table S3: Algae plastid genomes for brown algae phylogeny analysis

| ID | Organism | Class | Order | Family | Genus | Length |
| --- | --- | --- | --- | --- | --- | --- |
| MZ156041 | Desmarestia aculeata | Phaeophyceae | Desmarestiales | Desmarestiaceae | Desmarestia | 129228 |
| NC_036804 | Dictyopteris divaricata | Phaeophyceae | Dictyotales | Dictyotaceae | Dictyopteris | 126099 |
| NC_046005 | Cladosiphon okamuranus | Phaeophyceae | Ectocarpales | Chordariaceae | Cladosiphon | 137324 |
| NC_013498 | Ectocarpus siliculosus | Phaeophyceae | Ectocarpales | Ectocarpaceae | Ectocarpus | 139954 |
| MW762687 | Ishige okamurae | Phaeophyceae | Ishigeales | Ishigeaceae | Ishige | 129988 |
| NC_038231 | Endarachne binghamiae | Phaeophyceae | Ectocarpales | Scytosiphonaceae | Endarachne | 136274 |
| NC_044758 | Hapterophycus canaliculatus | Phaeophyceae | Ectocarpales | Scytosiphonaceae | Hapterophycus | 133508 |
| NC_057081 | Scytosiphon lomentaria | Phaeophyceae | Ectocarpales | Scytosiphonaceae | Scytosiphon | 134485 |
| NC_046447 | Scytosiphon promiscuus | Phaeophyceae | Ectocarpales | Scytosiphonaceae | Scytosiphon | 134358 |
| MG922855 | Fucus spiralis | Phaeophyceae | Fucales | Fucaceae | Fucus | 125066 |
| NC_016735 | Fucus vesiculosus | Phaeophyceae | Fucales | Fucaceae | Fucus | 124986 |
| NC_032288 | Coccophora langsdorfii | Phaeophyceae | Fucales | Sargassaceae | Coccophora | 124450 |
| MG459429 | Sargassum confusum | Phaeophyceae | Fucales | Sargassaceae | Sargassum | 124375 |
| MW784167 | Sargassum feldmannii | Phaeophyceae | Fucales | Sargassaceae | Sargassum | 124258 |
| NC_048511 | Sargassum fusiforme | Phaeophyceae | Fucales | Sargassaceae | Sargassum | 124298 |
| MW784163 | Sargassum graminifolium | Phaeophyceae | Fucales | Sargassaceae | Sargassum | 124563 |
| MT800998 | Sargassum hemiphyllum var. chinense | Phaeophyceae | Fucales | Sargassaceae | Sargassum | 124319 |
| MT873582 | Sargassum hemiphyllum var. chinense | Phaeophyceae | Fucales | Sargassaceae | Sargassum | 124323 |
| MW784169 | Sargassum henslowianum | Phaeophyceae | Fucales | Sargassaceae | Sargassum | 124450 |
| MT795188 | Sargassum horneri | Phaeophyceae | Fucales | Sargassaceae | Sargassum | 123982 |
| MT795189 | Sargassum horneri | Phaeophyceae | Fucales | Sargassaceae | Sargassum | 124053 |
| NC_029856 | Sargassum horneri | Phaeophyceae | Fucales | Sargassaceae | Sargassum | 124068 |
| MW767830 | Sargassum ilicifolium var. conduplicatum | Phaeophyceae | Fucales | Sargassaceae | Sargassum | 124414 |
| MW784168 | Sargassum mcclurei | Phaeophyceae | Fucales | Sargassaceae | Sargassum | 124272 |
| MW784166 | Sargassum muticum | Phaeophyceae | Fucales | Sargassaceae | Sargassum | 124401 |
| MW784165 | Sargassum phyllocystum | Phaeophyceae | Fucales | Sargassaceae | Sargassum | 124392 |
| NC_029134 | Sargassum thunbergii | Phaeophyceae | Fucales | Sargassaceae | Sargassum | 124592 |
| NC_028502 | Costaria costata | Phaeophyceae | Laminariales | Agaraceae | Costaria | 129947 |
| MZ156027 | Akkesiphycus lubricus | Phaeophyceae | Laminariales | Akkesiphycaceae | Akkesiphycus | 128910 |
| MT767060 | Alaria crispa | Phaeophyceae | Laminariales | Alariaceae | Alaria | 130587 |
| MT767062 | Alaria esculenta | Phaeophyceae | Laminariales | Alariaceae | Alaria | 130604 |
| MZ156044 | Alaria marginata | Phaeophyceae | Laminariales | Alariaceae | Alaria | 130568 |
| MZ156033 | Lessoniopsis littoralis | Phaeophyceae | Laminariales | Alariaceae | Lessoniopsis | 130839 |
| MZ156029 | Pterygophora californica | Phaeophyceae | Laminariales | Alariaceae | Pterygophora | 130581 |
| KU200463 | Undaria pinnatifida | Phaeophyceae | Laminariales | Alariaceae | Undaria | 130336 |
| NC_028503 | Undaria pinnatifida | Phaeophyceae | Laminariales | Alariaceae | Undaria | 130383 |
| MZ156037 | Chorda asiatica | Phaeophyceae | Laminariales | Chordaceae | Chorda | 130274 |
| MZ156043 | Arthrothamnus bifidus | Phaeophyceae | Laminariales | Laminariaceae | Arthrothamnus | 130498 |
| NC_044689 | Laminaria digitata | Phaeophyceae | Laminariales | Laminariaceae | Laminaria | 130377 |
| MZ156035 | Laminaria ephemera | Phaeophyceae | Laminariales | Laminariaceae | Laminaria | 130610 |
| NC_057231 | Laminaria rodriguezii | Phaeophyceae | Laminariales | Laminariaceae | Laminaria | 130541 |
| NC_044690 | Laminaria solidungula | Phaeophyceae | Laminariales | Laminariaceae | Laminaria | 130784 |
| MW899036 | Macrocystis integrifolia | Phaeophyceae | Laminariales | Laminariaceae | Macrocystis | 129320 |
| MZ156032 | Macrocystis pyrifera | Phaeophyceae | Laminariales | Laminariaceae | Macrocystis | 130196 |
| MZ156031 | Postelsia palmaeformis | Phaeophyceae | Laminariales | Laminariaceae | Postelsia | 129997 |
| NC_018523 | Saccharina japonica | Phaeophyceae | Laminariales | Laminariaceae | Saccharina | 130584 |
| NC_049039 | Saccharina latissima | Phaeophyceae | Laminariales | Laminariaceae | Saccharina | 130619 |
| MZ156036 | Saccharina subsessilis | Phaeophyceae | Laminariales | Laminariaceae | Saccharina | 130548 |
| MZ156038 | Ecklonia arborea | Phaeophyceae | Laminariales | Lessoniaceae | Ecklonia | 130965 |
| MZ156040 | Ecklonia radicosa | Phaeophyceae | Laminariales | Lessoniaceae | Ecklonia | 130860 |
| MZ156039 | Egregia menziesii | Phaeophyceae | Laminariales | Lessoniaceae | Egregia | 130044 |
| NC_056288 | Lessonia flavicans | Phaeophyceae | Laminariales | Lessoniaceae | Lessonia | 130085 |
| MZ156034 | Lessonia spicata | Phaeophyceae | Laminariales | Lessoniaceae | Lessonia | 130301 |
| NC_044182 | Lessonia spicata | Phaeophyceae | Laminariales | Lessoniaceae | Lessonia | 130305 |
| MZ156030 | Pseudochorda nagaii | Phaeophyceae | Laminariales | Pseudochordaceae | Pseudochorda | 129340 |

Figure S1:


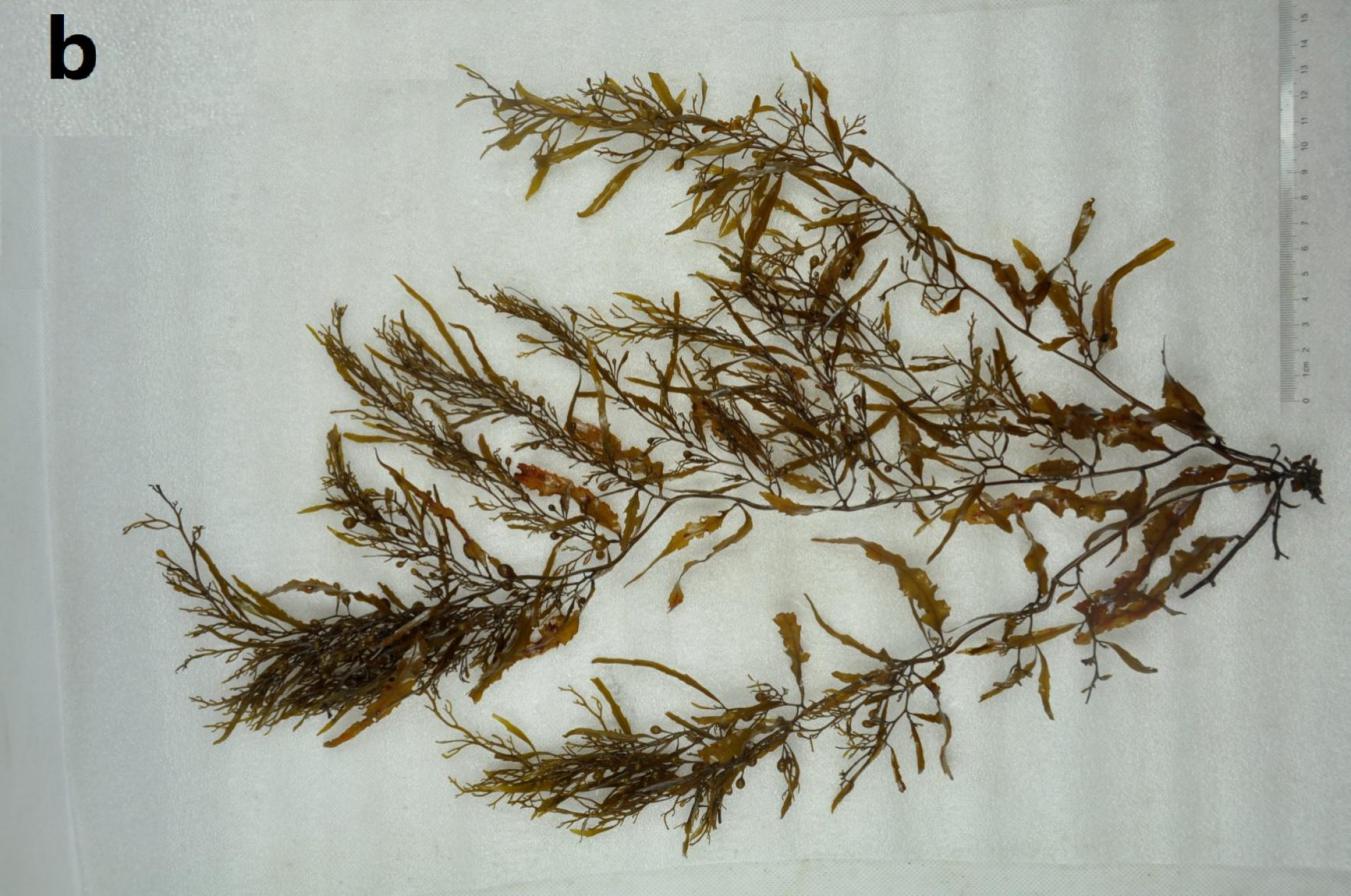

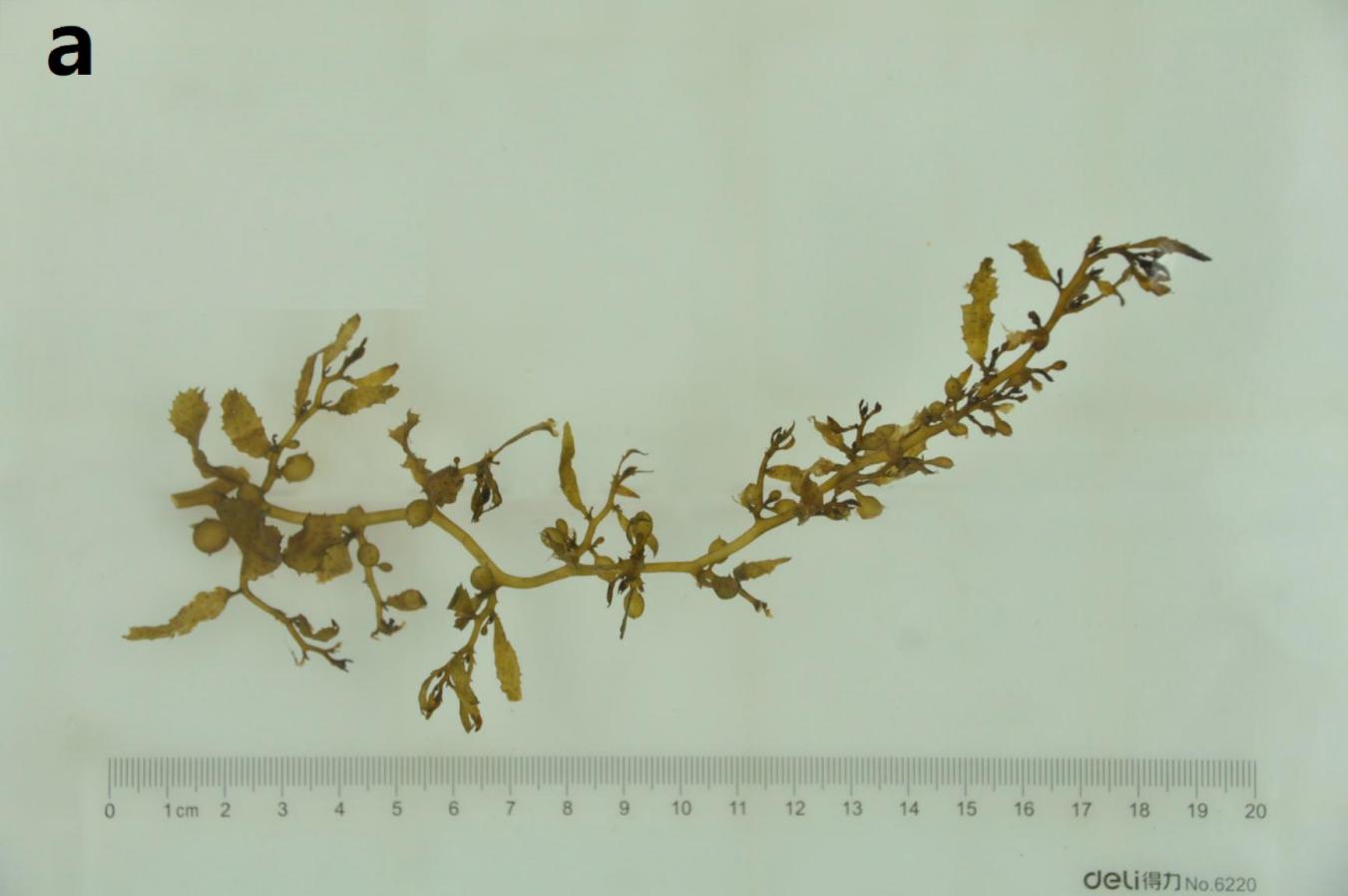


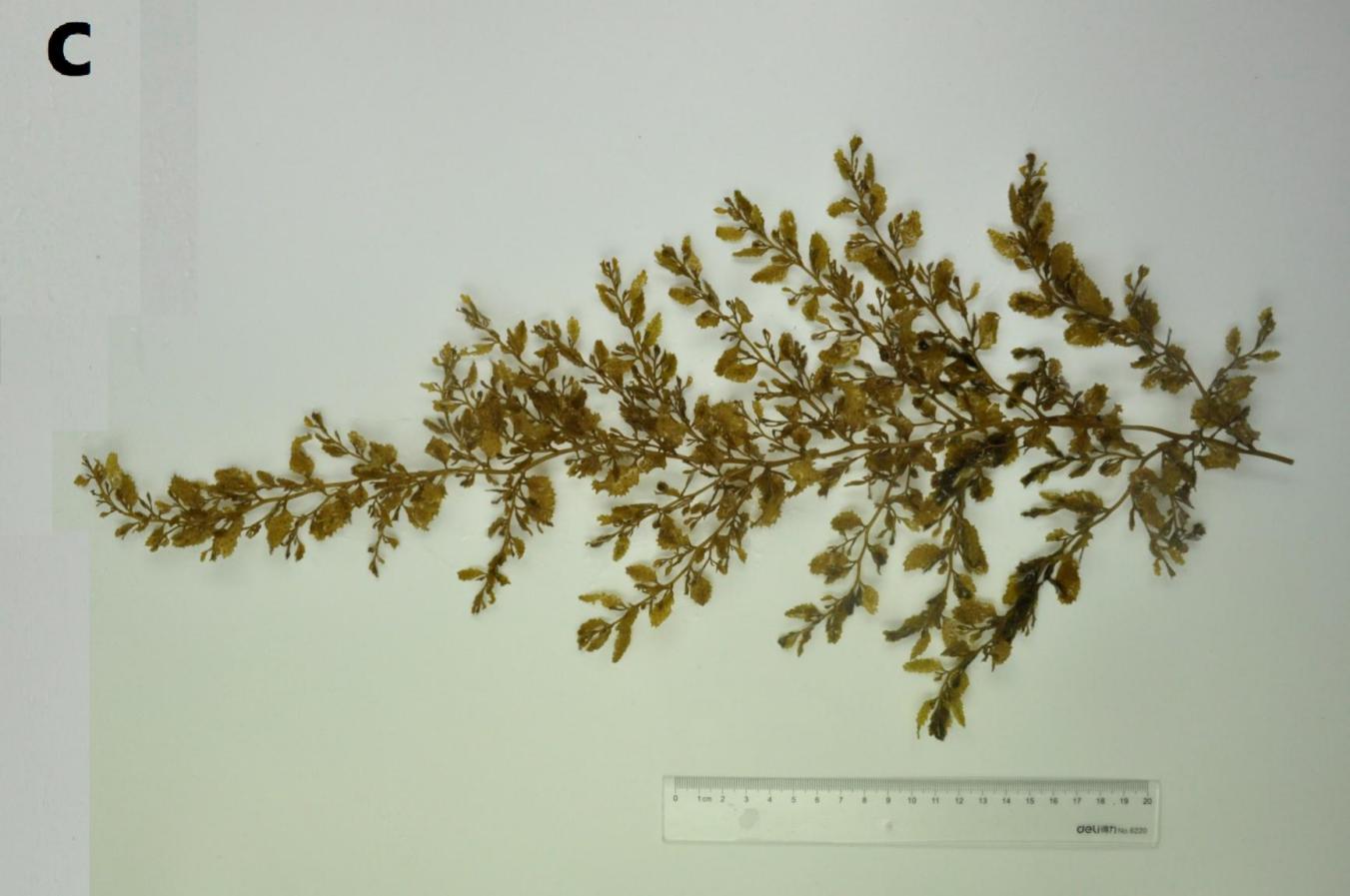


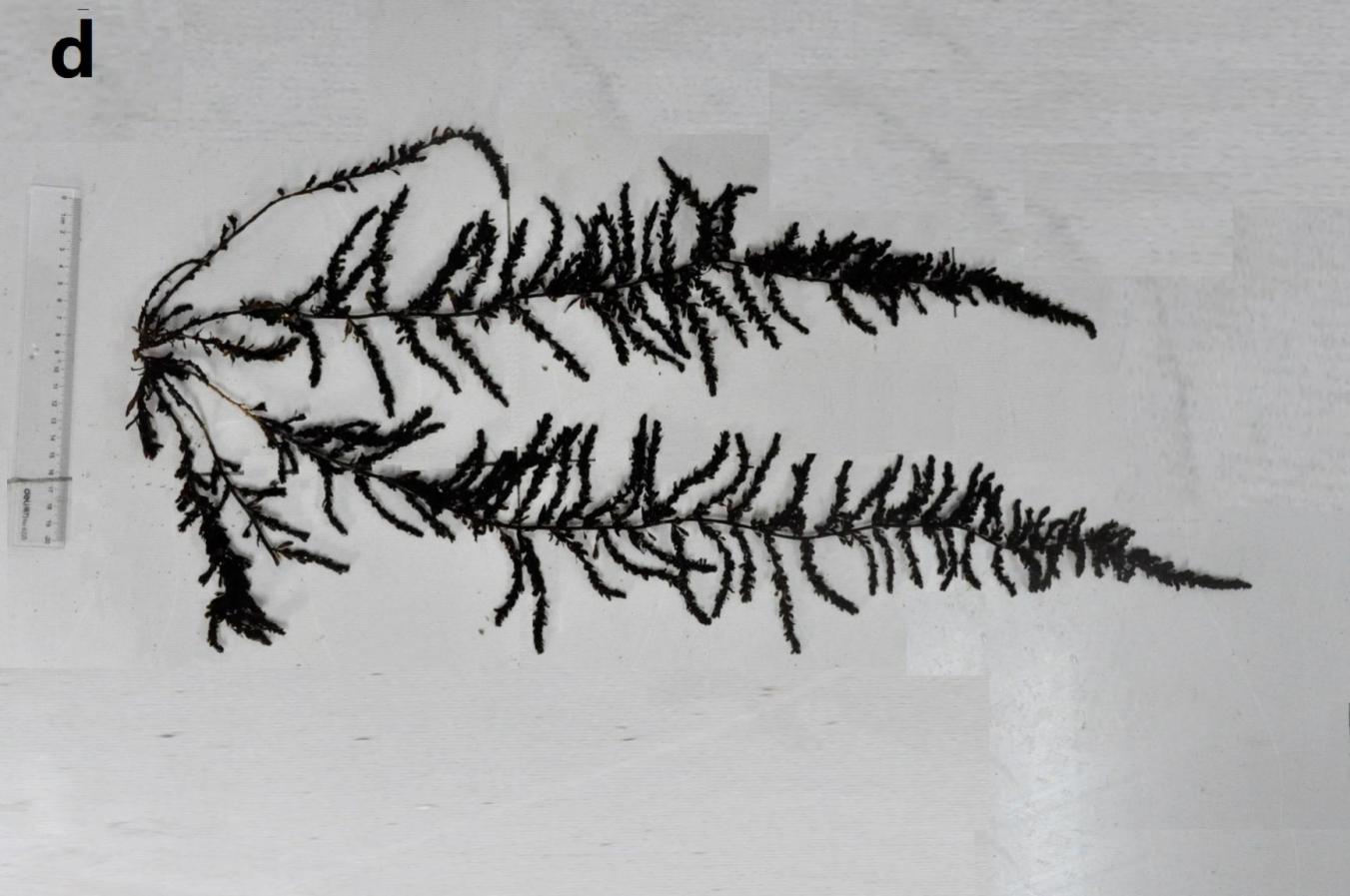


Figure S1(continued)


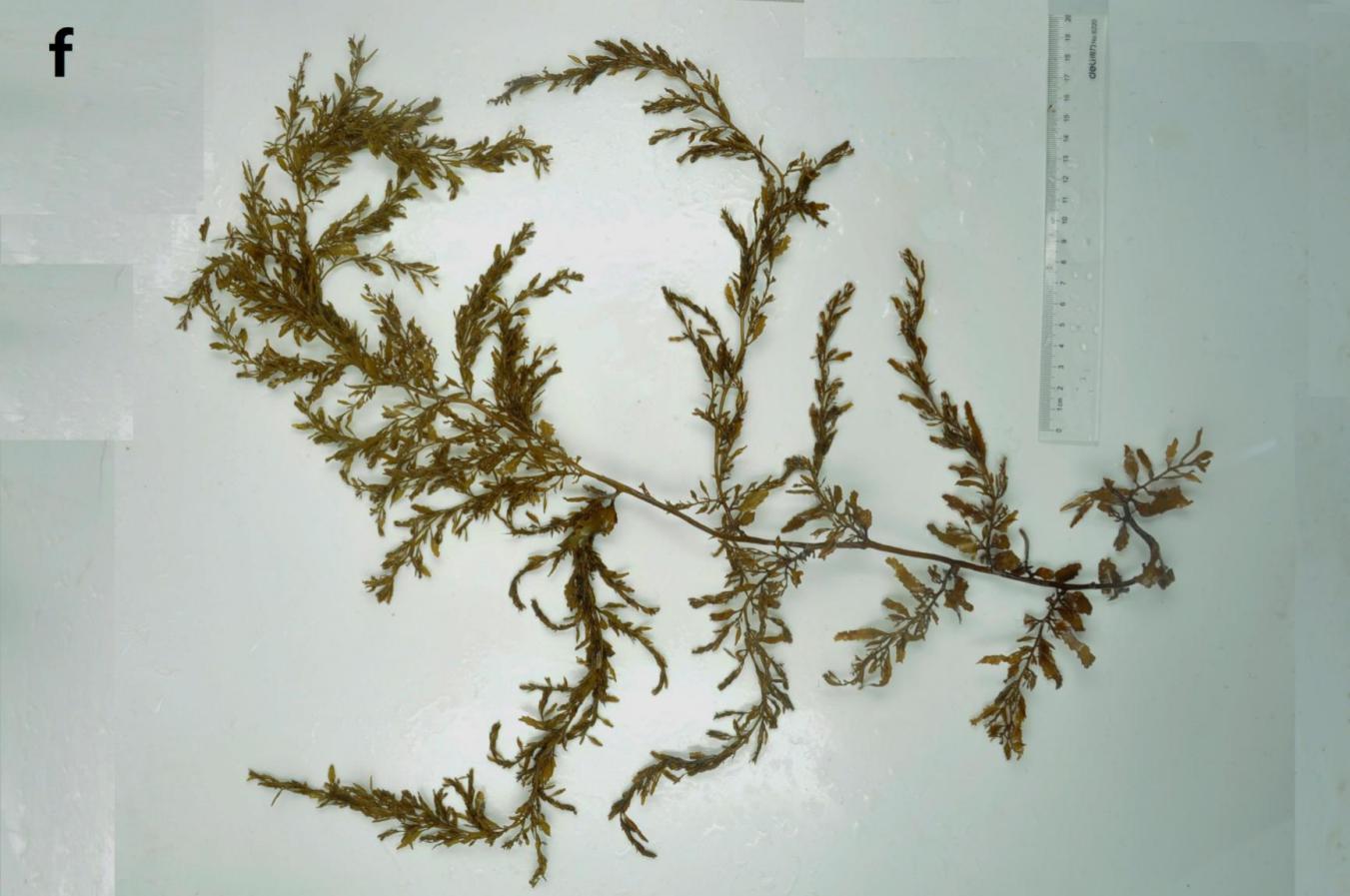

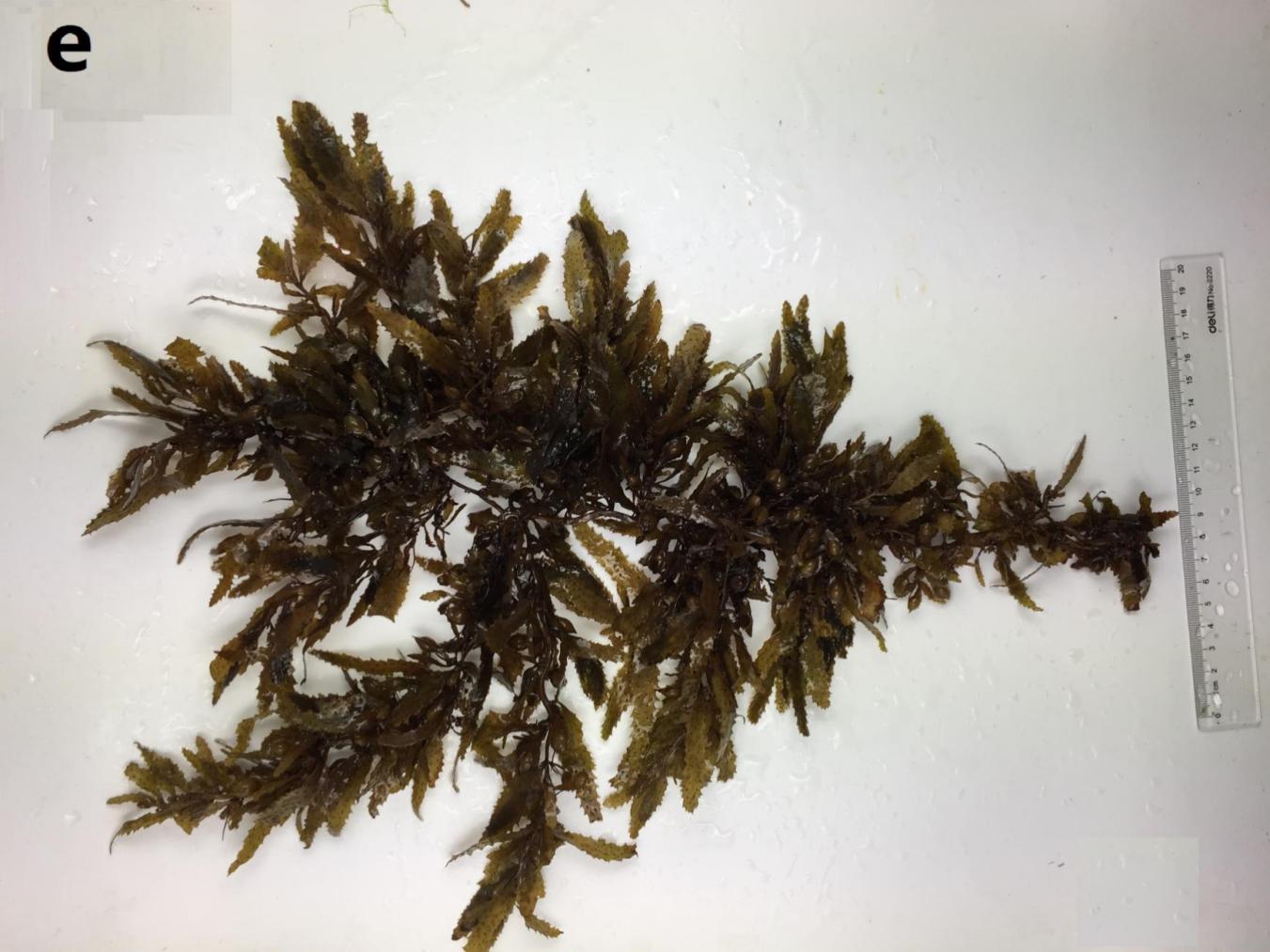


Figure S1(continued)


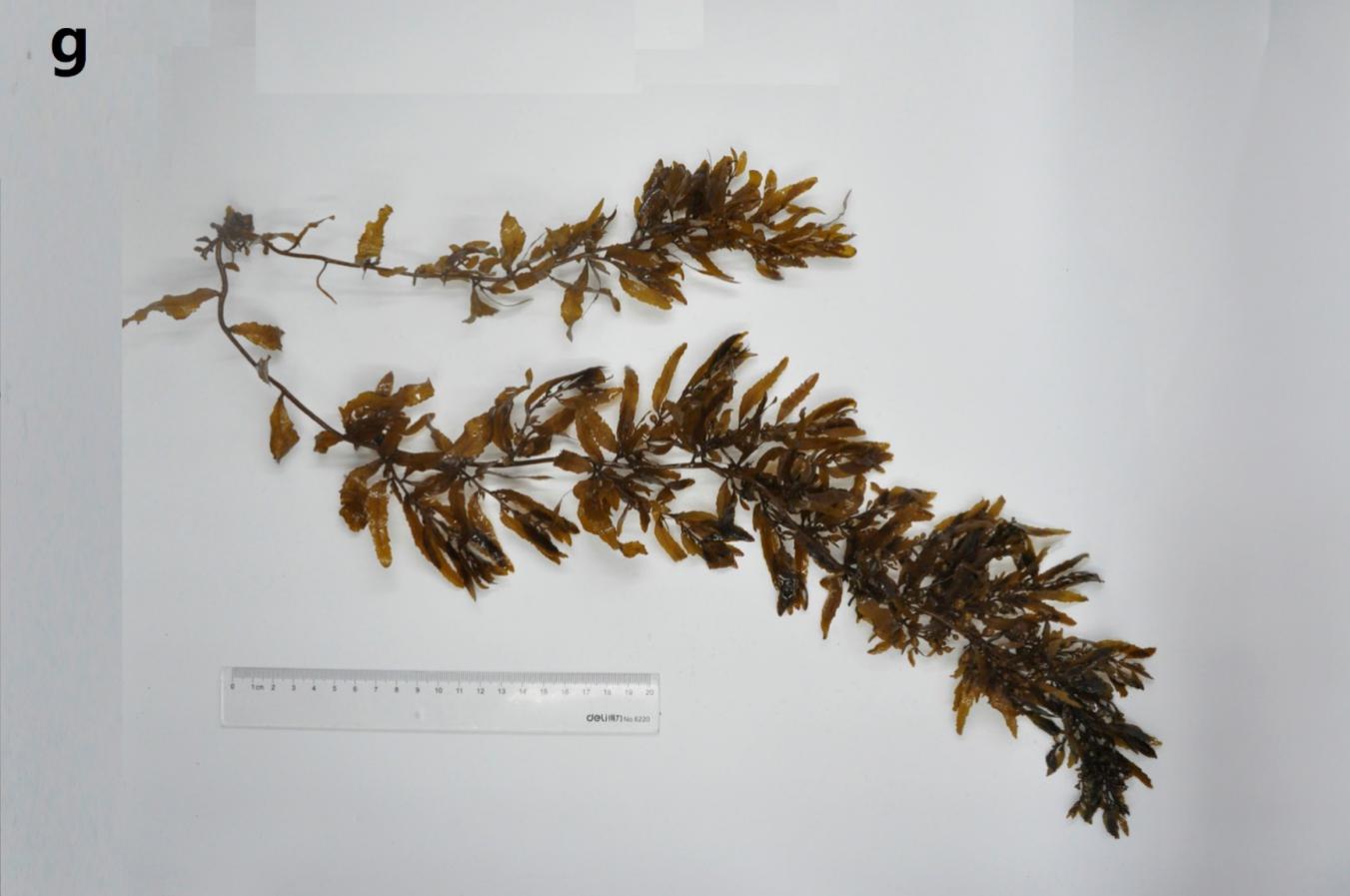
Figure S1(continued)

Sample biological characteristics of *S. ilicifolium var. conduplicatum* (a), *S. graminifolium* (b), *S. phyllocystum* (c), *S. muticum* (d), *S. feldmannii* (e), *S. mcclurei* (f) and *S. henslowianum* (g).

Figure S2:


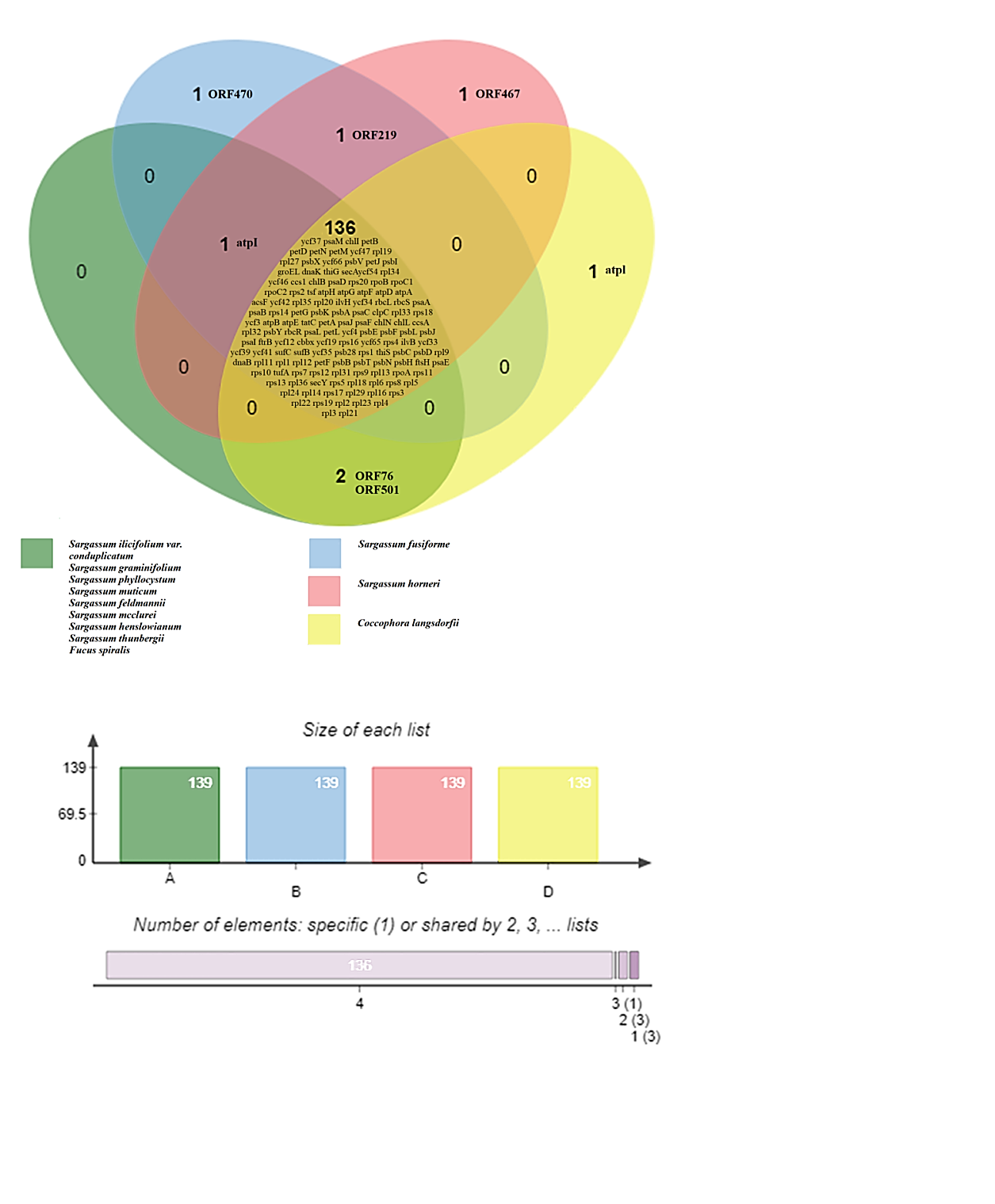
Venn diagram of protein-encoding gene content for the plastid genomes of twelve species in the order Fucales.
